# Macavirus latency-associated protein evades immune detection through regulation of protein synthesis *in cis* depending upon its glycin/glutamate-rich domain

**DOI:** 10.1371/journal.ppat.1006691

**Published:** 2017-10-23

**Authors:** Océane Sorel, Ting Chen, Françoise Myster, Justine Javaux, Alain Vanderplasschen, Benjamin G. Dewals

**Affiliations:** Immunology-Vaccinology, Department of infectious and parasitic diseases, Faculty of Veterinary medicine–FARAH, University of Liège, Liège, Belgium; University of North Carolina at Chapel Hill, UNITED STATES

## Abstract

Alcelaphine herpesvirus 1 (AlHV-1) is a γ-herpesvirus (γ-HV) belonging to the macavirus genus that persistently infects its natural host, the wildebeest, without inducing any clinical sign. However, cross-transmission to other ruminant species causes a deadly lymphoproliferative disease named malignant catarrhal fever (MCF). AlHV-1 ORF73 encodes the latency-associated nuclear antigen (LANA)-homolog protein (aLANA). Recently, aLANA has been shown to be essential for viral persistence *in vivo* and induction of MCF, suggesting that aLANA shares key properties of other γ-HV genome maintenance proteins. Here we have investigated the evasion of the immune response by aLANA. We found that a glycin/glutamate (GE)-rich repeat domain was sufficient to inhibit *in cis* the presentation of an epitope linked to aLANA. Although antigen presentation in absence of GE was dependent upon proteasomal degradation of aLANA, a lack of GE did not affect protein turnover. However, protein self-synthesis *de novo* was downregulated by aLANA GE, a mechanism directly associated with reduced antigen presentation *in vitro*. Importantly, codon-modification of aLANA GE resulted in increased antigen presentation *in vitro* and enhanced induction of antigen-specific CD8^+^ T cell responses *in vivo*, indicating that mRNA constraints in GE rather than peptidic sequence are responsible for *cis*-limitation of antigen presentation. Nonetheless, GE-mediated limitation of antigen presentation *in cis* of aLANA was dispensable during MCF as rabbits developed the disease after virus infection irrespective of the expression of full-length or GE-deficient aLANA. Altogether, we provide evidence that inhibition *in cis* of protein synthesis through GE is likely involved in long-term immune evasion of AlHV-1 latent persistence in the wildebeest natural host, but dispensable in MCF pathogenesis.

## Author summary

The macavirus alcelaphine herpesvirus 1 (AlHV-1) is a γ-herpesvirus (γ-HV) that has been first isolated in East-Africa in 1960 from a wildebeest and identified to be the etiological agent of malignant catarrhal fever (MCF) in bovine. An interesting aspect of AlHV-1 is that it can persist in wildebeest by remaining latent whereas it induces MCF upon cross-species transmission in several species of ruminants including cattle. MCF is a deadly lymphoproliferative disease developing after a prolonged incubation period. We have recently demonstrated that viral genome maintenance by the latency-associated viral protein aLANA is essential for inducing MCF. In the present study, we have investigated the ability of aLANA to evade antigen-specific cytotoxic T lymphocytes, an important property of γ-HV genome maintenance proteins to enable long term virus persistence. We provide evidence that GE, a specific repeated region in the nucleotidic sequence of aLANA, is directly involved in restraining aLANA protein synthesis. Although the lack of GE in aLANA did not significantly affect MCF induction in the rabbit model, such mechanism resulted in severely reduced presentation of an antigenic peptide linked to aLANA in a model *in vitro* and ineffective induction of antigen-specific CD8^+^ T lymphocyte responses *in vivo*. These results are important as they suggest that immune evasion of aLANA during MCF in susceptible species is not essential in the pathogenesis of the disease but identified GE to be essential for immune evasion during latency, likely in the wildebeest natural host.

## Introduction

Infection with the members of the *Herpesviridae* family is characterized with lifelong persistence of the viral genomes in the infected hosts. Such latent infection is only possible through the establishment of complex immune evasion mechanisms. Latent infection by γ-herpesviruses (γ-HV) is mostly asymptomatic and mainly occurs in lymphoid cells. However, γ-HV latency can induce lymphoproliferative diseases and cancers. As such, Epstein-Barr virus (EBV) infection of immunocompromised human individuals has been associated with nasopharyngeal carcinoma, Burkitt’s and Hodgkin’s lymphomas and Kaposi’s sarcoma associated-herpesvirus (KSHV) has been associated with primary effusion lymphomas, Castleman’s disease and Kaposi’s sarcoma [1–3]. In addition, lymphoproliferative diseases are caused by other γ-HVs in specific cases of cross-species transmission [4, 5]. The development of such malignancies has been related to γ-HV latent infection. Latent infection of host cells by many γ-HVs is dependent upon the expression of a viral genome maintenance protein (GMP), which ensures the persistence of the viral episome within actively dividing cells, yet simultaneously evades the immune surveillance. Interestingly, specific EBNA1 and LANA1 peptides have been shown to be presented by cross-priming and specific CD8^+^ T cells could be readily isolated from EBV- or KSHV-infected individuals [6–8]. In addition, recent reports investigating the immune evasion mechanisms by γ-HV GMPs suggest that latently infected cells evade the detection by host CD8^+^ cytotoxic T lymphocytes (CTLs) rather by a limitation of antigen presentation than an absence of T cell epitopes.

Malignant catarrhal fever (MCF) is an acute, sporadic and fatal pan-systemic lymphoproliferative disease of a variety of species of the *Artiodactyla* order, including cattle. The main causative agents of MCF are two γ-HVs that are grouped in the macavirus genus, ovine herpesvirus 2 (OvHV-2) and alcelaphine herpesvirus 1 (AlHV-1). These viruses cause no apparent disease in their natural host species. Sheep are naturally infected by OvHV-2, which is responsible for the sporadic sheep-associated form of MCF [5]. Wildebeest are persistently infected with AlHV-1, the causative agent of the wildebeest-derived form of the disease [9, 10]. The prevalence of AlHV-1 infection in wildebeest is close to 100% and transmission to MCF-susceptible species mainly occurs during the calving period and in the first months of life [11, 12]. MCF impact on the local pastoralist populations has largely been underestimated, with recent reports demonstrating that MCF is perceived to be the cattle disease with the highest economic and social impacts in these areas [13–16]. In addition, MCF has been reported throughout the world in game farms or zoological collections where mixed ruminant species including wildebeest are kept [17].

Recent data demonstrated that MCF is caused by the activation and proliferation of latently infected CD8^+^ T cells [18–20] and that the expression of the AlHV-1 genome maintenance protein, the latency-associated nuclear antigen (LANA)-homolog (aLANA) encoded by the ORF73 gene of AlHV-1 is essential for both viral persistence in infected hosts and induction of MCF [21]. The expression of aLANA is abundant in the tissues of MCF-developing animals and should potentially induce an anti-viral cytotoxic response. Although such anti-viral response might exist, it is however not protective. Many γ-HVs genome maintenance proteins have *cis*-acting immune evasion mechanisms mainly, but not only, regulated by their large acidic repeat domains [22–29]. A common feature of γ-HV GMPs is their evasion of specific CTLs through *cis-*acting but diverse mechanisms [30–34].

Here we have investigated the immune evasion properties of aLANA. We showed that a glycin/glutamate (GE)-rich repeat domain in aLANA was necessary and sufficient to inhibit the presentation by MHC-I of an epitope linked to it. We further found that aLANA GE downregulated protein self-synthesis and this mechanism could be associated with reduced antigen presentation *in vitro*. Importantly, codon modification of the purine bias in GE resulted in heightened antigen presentation of an epitope linked to aLANA and heightened induction of antigen-specific CD8^+^ T cell responses. These results suggested that inhibition *in cis* of antigen presentation is controlled by mRNA constraints in GE and reduced protein translation efficiency. Nonetheless, infection of rabbits with an AlHV-1 recombinant strain expressing a GE-deficient aLANA protein did not affect MCF induction, suggesting a dispensable role of *cis-*acting immune evasion by aLANA in the pathogenesis of MCF. These findings enlarge our understanding of MCF pathogenesis and suggest that *cis-*acting immune evasion by aLANA is likely involved in long-term viral persistence in latently infected wildebeests.

## Results

### Limited presentation of a CD8^+^ T cell epitope linked in *cis* to alcelaphine herpesvirus 1 aLANA

**Fig 1:**
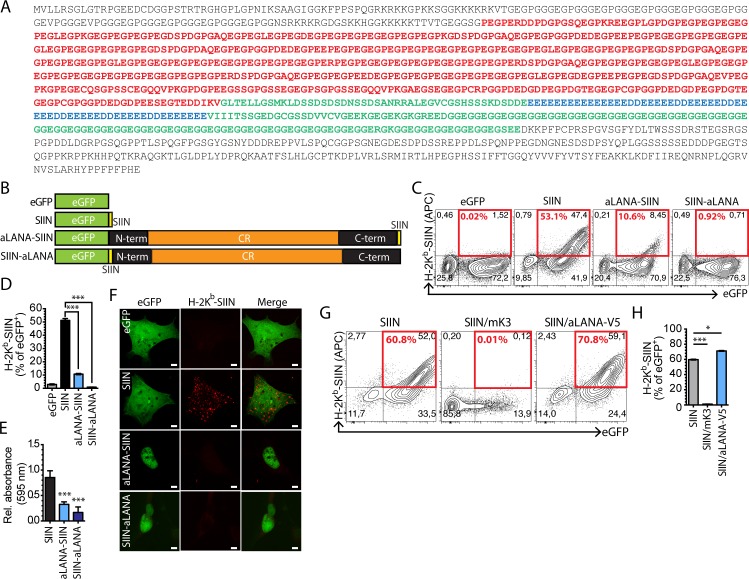
*Cis*-acting inhibition of antigen presentation of aLANA. (A) Peptidic coding sequence of aLANA. The acidic central repeat region is highlighted. Residues in red represent the region rich in glycin, glutamate, and proline, consequently termed GPE. Residues in blue represent the region rich in glutamate consequently named E. Residues in green represent the region rich in glycin and glutamate and consequently named GE. The GE-rich domain comprises the E-rich region. N- and C-terminal regions of aLANA are in black. (B) Schematic description of expression constructs. The expression plasmid peGFP-C1 (eGFP) was used to fuse a H-2K^b^-restricted ovalbumin CTL epitope, SIINFEKL, in-frame to a C-terminal enhanced Green Fluorescent Protein (eGFP-SIIN). The aLANA coding sequence was then inserted in-frame to the eGFP-SIIN plasmid leaving the SIINFEKL in the C-terminus end of aLANA (aLANA-SIIN) or in the N-terminus end of aLANA (SIIN-aLANA) to allow analysis of endogenous processing of aLANA. (C) Representative results from flow cytometry analysis of SIINFEKL presentation on 293Kb cells. Cells were stained with APC-conjugated anti-mouse H-2K^b^-SIINFEKL complex (25-D1.16) 48h after transfection with the indicated constructs. Numbers in red boxes indicate percent of H-2K^b^-SIINFEKL-positive cells within eGFP^+^ cells. (D) Percent of eGFP^+^ cells expressing H-2K^b^-SIINFEKL complexes at the cell surface based on the analysis in C. (E) The SIINFEKL-specific T cell hybridoma B3Z was co-cultured overnight with 293Kb cells 48h after transfection with the different constructs. *β*-galactosidase activity was assayed by cell lysis in the presence of chlorophenol-red-*β*-D-galactoside (CPRG) and absorbance read at 595 nm. Values relative to eGFP negative construct are shown. (F) Confocal analysis of cell surface expression of H-2K^b^-SIINFEKL expression 48h after transfection of 293Kb cells with the different constructs. Scale bar = 5 μm. (G) Representative results from flow cytometry analysis of SIINFEKL presentation on 293Kb cells 48h after co-transfection with the SIIN construct and plasmids expressing MuHV-4 K3 protein (mK3) that degrades MHC class I heavy chains, or a aLANA-V5 fusion protein [21]. (H) Percent of eGFP^+^ cells expressing H-2K^b^-SIINFEKL complexes at the cell surface based on the analysis in G. Bars show mean ± S.D. (n = 3). Statistical analyses by one-way ANOVA and Dunnett’s post-test with SIIN as control comparison mean (***p≤0.001).

Sequencing of the AlHV-1 genome revealed that its ORF73 gene encodes a 1300 amino acid-long protein [35], which makes AlHV-1 LANA-homolog protein (aLANA) the longest GMP ortholog described to date among rhadinoviruses and macaviruses. Analysis of the primary structure of aLANA revealed the presence of a long central repeat (CR) region that could be divided in two main subregions based on the abundance of glycine, proline and glutamate (GPE-rich region) or glycine and glutamate residues (GE-rich region). An additional region containing glutamate repeats is enclosed within the GE-rich region, and was termed the E-rich region (Fig 1A). We hypothesized that aLANA possesses mechanisms inhibiting antigen presentation *in cis* similar to other γ-HV GMPs and therefore renders AlHV-1 latently infected cells difficult to detect by cytotoxic T cells. We used the SIINFEKL peptide of chicken ovalbumin as cognate epitope to be linked in frame to eGFP and transient expression in 293Kb cells for detection of H-2Kb-peptide complexes [36]. In this model, 293Kb cells constitutively express the murine H-2Kb MHC class I haplotype. 293Db cells were used as controls to determine the specificity of the staining (S1A Fig). In order to determine the capability of aLANA to inhibit antigen presentation *in cis*, we introduced AlHV-1 ORF73 coding sequence into the peGFP-SIIN plasmid to generate vectors expressing aLANA-SIIN and SIIN-aLANA proteins (Fig 1B). 293Kb cells transiently expressing aLANA-SIIN or SIIN-LANA expressed very low amounts of MHC-epitope complexes at the cell surface and were poorly recognized by SIINFEKL-specific B3Z hybridoma cells (Fig 1C–1F). Similar results were also obtained using L929Kb and VeroKb cell lines (S1B and S1C Fig). These results suggested poor antigen processing. There was no evidence for aLANA inhibiting peptide presentation from co-transfected eGFP-SIIN (Fig 1G and 1H). These results suggested an immune evasion mechanism by aLANA acting *in cis* and not *in trans*.

### The central repeat region of aLANA is involved in *cis*-acting inhibition of antigen presentation

**Fig 2:**
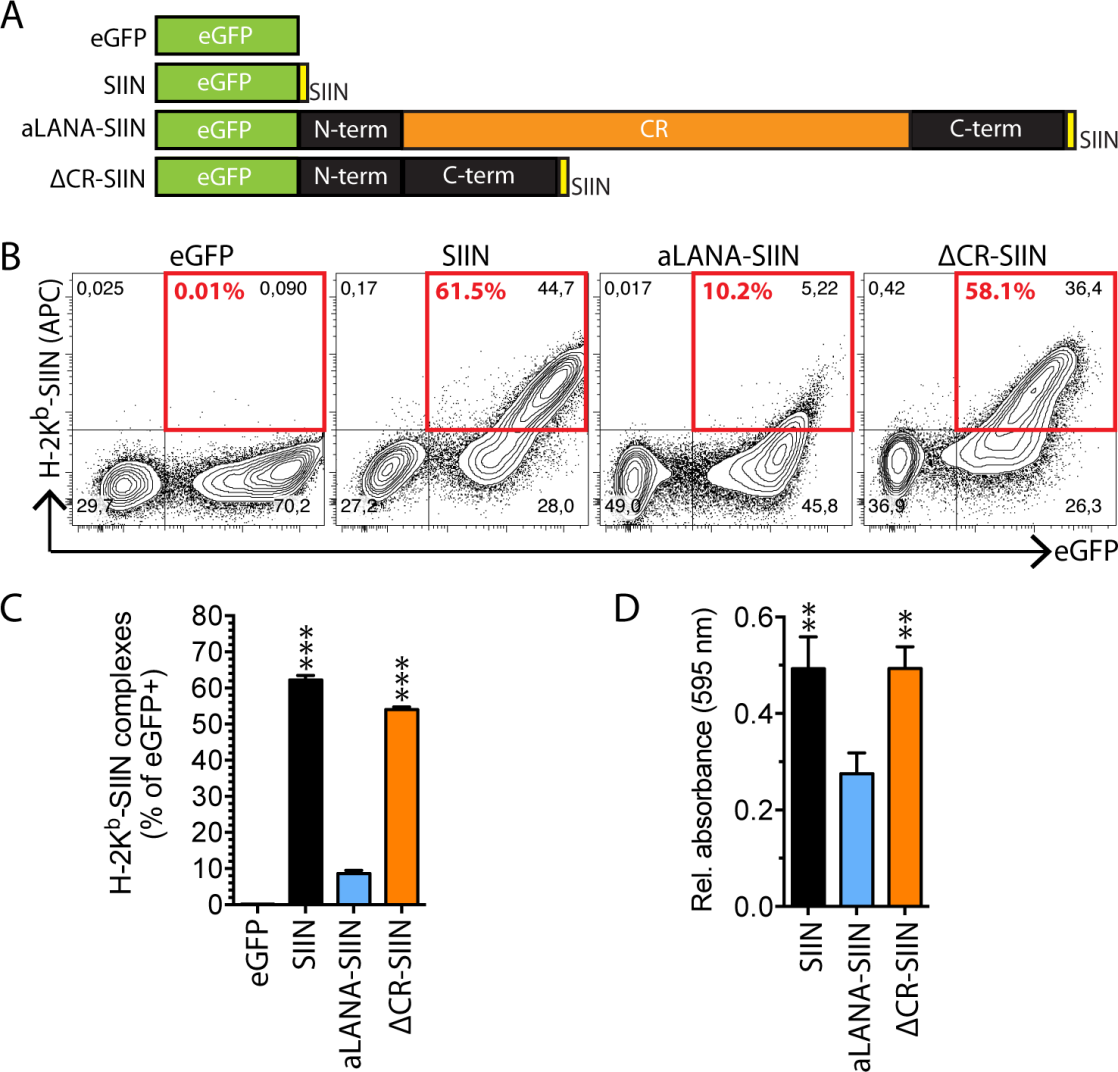
Deletion of the central repeat region of aLANA restores OVA-peptide presentation. (A) Schematic description of aLANA expression constructs. The full central repeat region was truncated from aLANA to generate a ΔCR-SIIN construct. (B) Representative results from flow cytometry analysis of SIINFEKL presentation on 293Kb cells 48h after transfection with the indicated constructs. Numbers in red boxes indicate percent of H-2K^b^-SIINFEKL-positive cells within eGFP^+^ cells. (C) Percent of eGFP^+^ cells expressing H-2K^b^-SIINFEKL complexes at the cell surface based on the analysis in B. (D) *β*-galactosidase activity in B3Z after overnight co-culture with 293Kb cells 48h after transfection with the indicated constructs. Bars show mean ± S.D. (n = 3). Statistical analyses by one-way ANOVA and Dunnett’s post-test with aLANA-SIIN as control comparison mean (**p≤0.01, ***p≤0.001).

To identify possible contributions of the CR region in aLANA reduced antigen presentation, we constructed a mutant form of aLANA from which the entire CR domain was removed. The resulting ΔCR aLANA mutant protein was inserted into the peGFP-SIIN expression vector in order to express the ΔCR-SIIN protein (Fig 2A). The expression of MHC-epitope complexes at the cell surface of 293Kb cells transiently expressing ΔCR-SIIN was rescued to levels similar to the SIIN positive control (Fig 2B and 2C). ΔCR-SIIN expression was restricted to the nuclei like the full-length form of aLANA (S2 Fig). Also, 293Kb cells expressing ΔCR-SIIN or SIIN induced similar levels of β-galactosidase activity in B3Z (Fig 2D). These results suggested that antigen processing of aLANA is impaired by the presence of the CR region.

### A key region of aLANA for *cis*-acting inhibition of MHC class I-restricted epitope presentation

**Fig 3:**
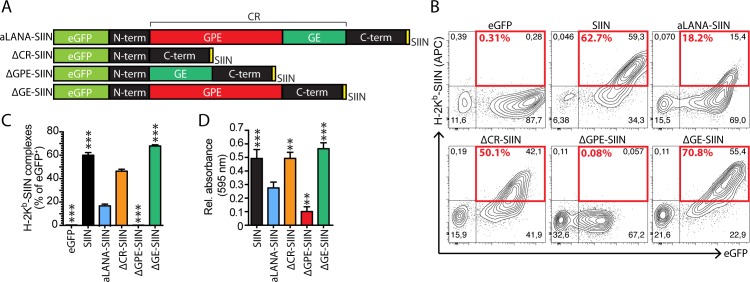
Deletion of the GE-rich domain of aLANA restores OVA-peptide presentation. (A) Schematic description of the aLANA expression constructs deleted for the GPE or GE-rich domains, to generate the ΔGPE-SIIN and ΔGE-SIIN constructs. (B) Representative results from flow cytometry analysis of SIINFEKL presentation on 293Kb cells 48h after transfection with the indicated constructs. Numbers in red boxes indicate percent of H-2K^b^-SIINFEKL-positive cells within eGFP^+^ cells. (C) Percent of eGFP^+^ cells expressing H-2K^b^-SIINFEKL complexes at the cell surface based on the analysis in B. (D) *β*-galactosidase activity in B3Z after overnight co-culture with 293Kb cells 48h after transfection with the indicated constructs. Bars show mean ± S.D. (n = 3). Statistical analyses by one-way ANOVA and Dunnett’s post-test with aLANA-SIIN as control comparison mean (**p≤0.01, ***p≤0.001).

**Fig 4:**
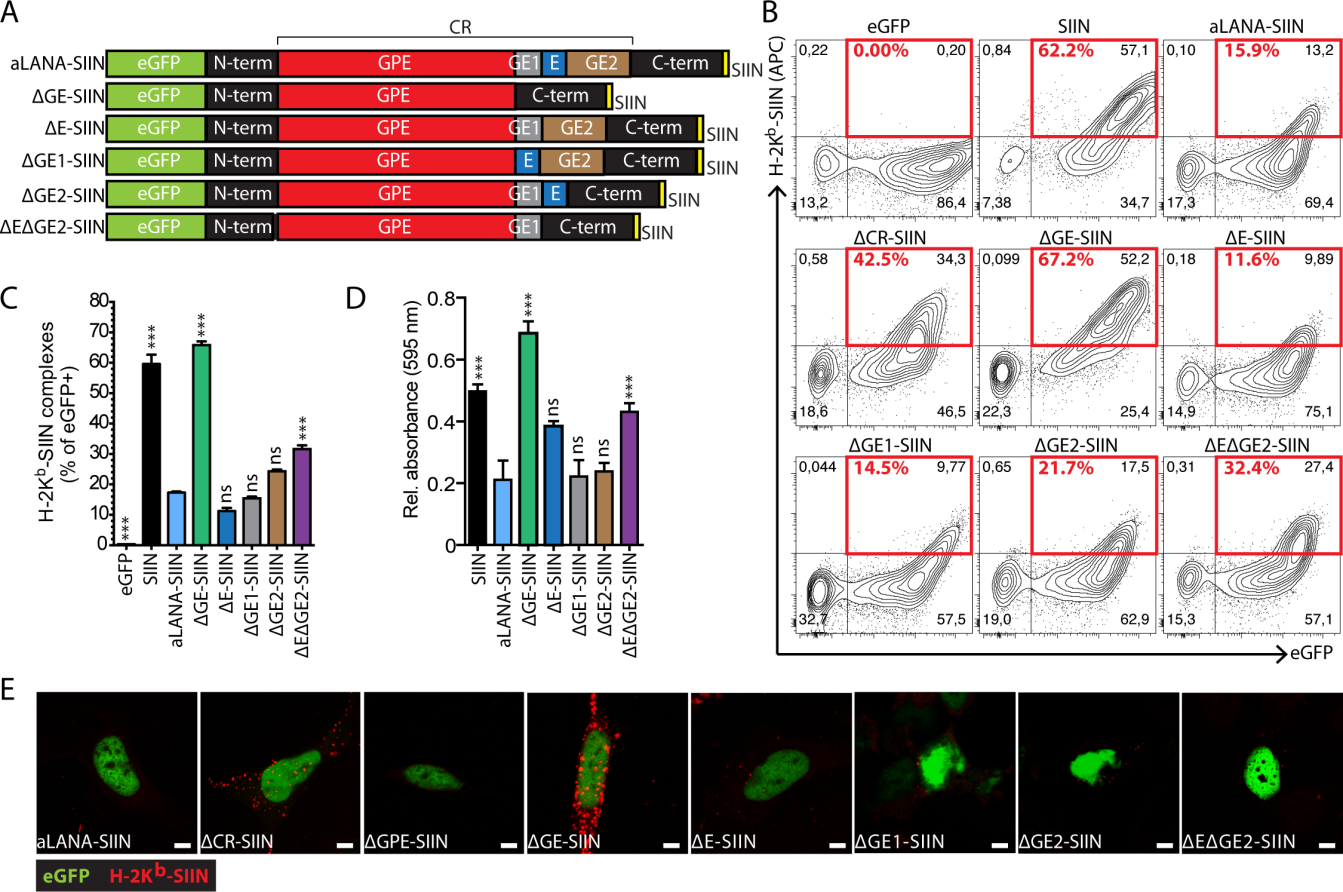
The entire GE-rich domain is necessary to inhibit the presentation of a linked-peptide. (A) Schematic description of the aLANA expression constructs deleted for the E, GE1 and GE2 domains, to generate the ΔE-SIIN, ΔGE1-SIIN, ΔGE2-SIIN, and ΔEΔGE2-SIIN constructs. (B) Representative results from flow cytometry analysis of SIINFEKL presentation on 293Kb cells 48h after transfection with the indicated constructs. Numbers in red boxes indicate percent of H-2K^b^-SIINFEKL-positive cells within eGFP^+^ cells. (C) Percent of eGFP^+^ cells expressing H-2K^b^-SIINFEKL complexes at the cell surface based on the analysis in B. (D) *β*-galactosidase activity in B3Z after overnight co-culture with 293Kb cells 48h after transfection with the indicated constructs. (E) Confocal analysis of cell surface expression of H-2K^b^-SIINFEKL expression 48h after transfection of 293Kb cells with the different constructs. Scale bar = 5 μm. Bars show mean ± S.D. (n = 3). Statistical analyses by one-way ANOVA and Dunnett’s post-test with aLANA-SIIN as control comparison mean (***p≤0.001).

Next, we investigated potential contributions of the subregions of the CR domain in limiting antigen presentation *in cis*. First, we produced two forms of the aLANA protein lacking either the GPE domain (ΔGPE-SIIN) or the purine-enriched GE domain (ΔGE-SIIN), respectively (Fig 3A). 293Kb cells expressing ΔGPE-SIIN showed intranuclear localization of eGFP expression (S2 Fig) and strongly reduced antigen presentation at the cell surface as well as reduced B3Z activation (Fig 3B–3D). ΔGE-SIIN expression in 293Kb cells was also intranuclear (S2 Fig), but strikingly resulted in rescued peptide presentation and activation of B3Z cells to levels similar to the SIIN control (Fig 3B–3D). Thus, the GE subregion of aLANA impaired antigen processing and presentation of a peptide linked to it. Supporting this conclusion, similar results could be observed in VeroKb cells (S1C Fig) and also using plasmid vectors in which protein expression is driven by an eukaryotic EF1-α promoter (S1D Fig). Next, we investigated the role of the E-rich domain within the GE region of aLANA as well as both flanking subregions named GE1 and GE2, respectively (Fig 4A). We produced four additional constructs expressing different combinations of the GE subregions, namely ΔE-, ΔGE1-, ΔGE2-, and ΔEΔGE2-SIIN. Expression of these mutant forms of aLANA in 293Kb cells did not reach the levels of antigen presentation obtained with ΔGE-SIIN (Fig 4B–4E), suggesting that the entire GE region is necessary for limiting antigen presentation *in cis*.

### aLANA GE-rich domain is sufficient for limiting antigen presentation *in cis*

**Fig 5:**
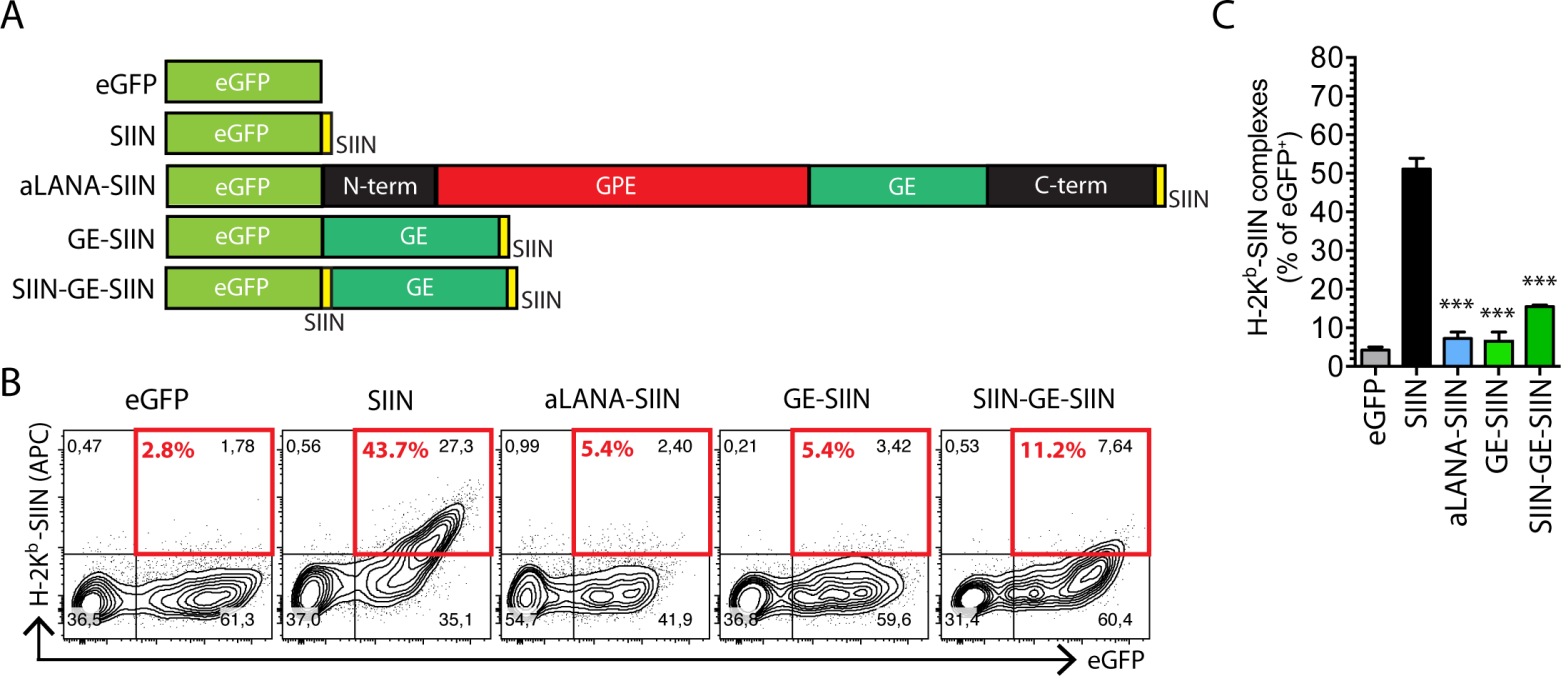
The GE-rich domain of aLANA is sufficient to regulate OVA-peptide presentation. (A) Schematic representation of peGFP-C1 expression constructs to generate the GE-SIIN and SIIN-GE-SIIN expression constructs. The GE-rich domain was inserted in-frame between the eGFP coding sequence and the SIINFEKL tag (GE-SIIN) followed by insertion of an additional SIINFEKL tag at the N-terminus end of the GE sequence. (B) Representative results from flow cytometry analysis of SIINFEKL presentation on 293Kb cells 48h after transfection with the indicated constructs. Numbers in red boxes indicate percent of H-2K^b^-SIINFEKL-positive cells within eGFP^+^ cells. (C) Percent of eGFP^+^ cells expressing H2-K^b^-SIINFEKL complexes at the cell surface based on the analysis in B. Bars show mean ± S.D. (n = 3). Statistical analyses by one-way ANOVA and Dunnett’s post-test with SIIN as control comparison mean (*p≤0.05, ***p≤0.001).

To examine the possibility that the GE-rich domain is self-sufficient to inhibit the presentation of a linked epitope, we expressed a chimeric protein composed in N-terminus of the eGFP coding sequence fused with the GE domain and a C-terminal SIINFEKL epitope tag (Fig 5A, GE-SIIN). To further investigate if the position of the SIINFEKL peptide could affect its presentation, another construct consisted in the insertion of an additional SIINFEKL epitope tag in between eGFP and GE (Fig 5A, SIIN-GE-SIIN). Following transfection of 293Kb cells, expression of both GE-SIIN and SIIN-GE-SIIN could be detected in both cytoplasm and nucleus and antigen presentation was strongly limited by the presence of the GE domain (Fig 5B and 5C). These results demonstrated that the GE domain is sufficient to significantly reduce antigen presentation.

### Proteasomal degradation of aLANA and turnover

**Fig 6:**
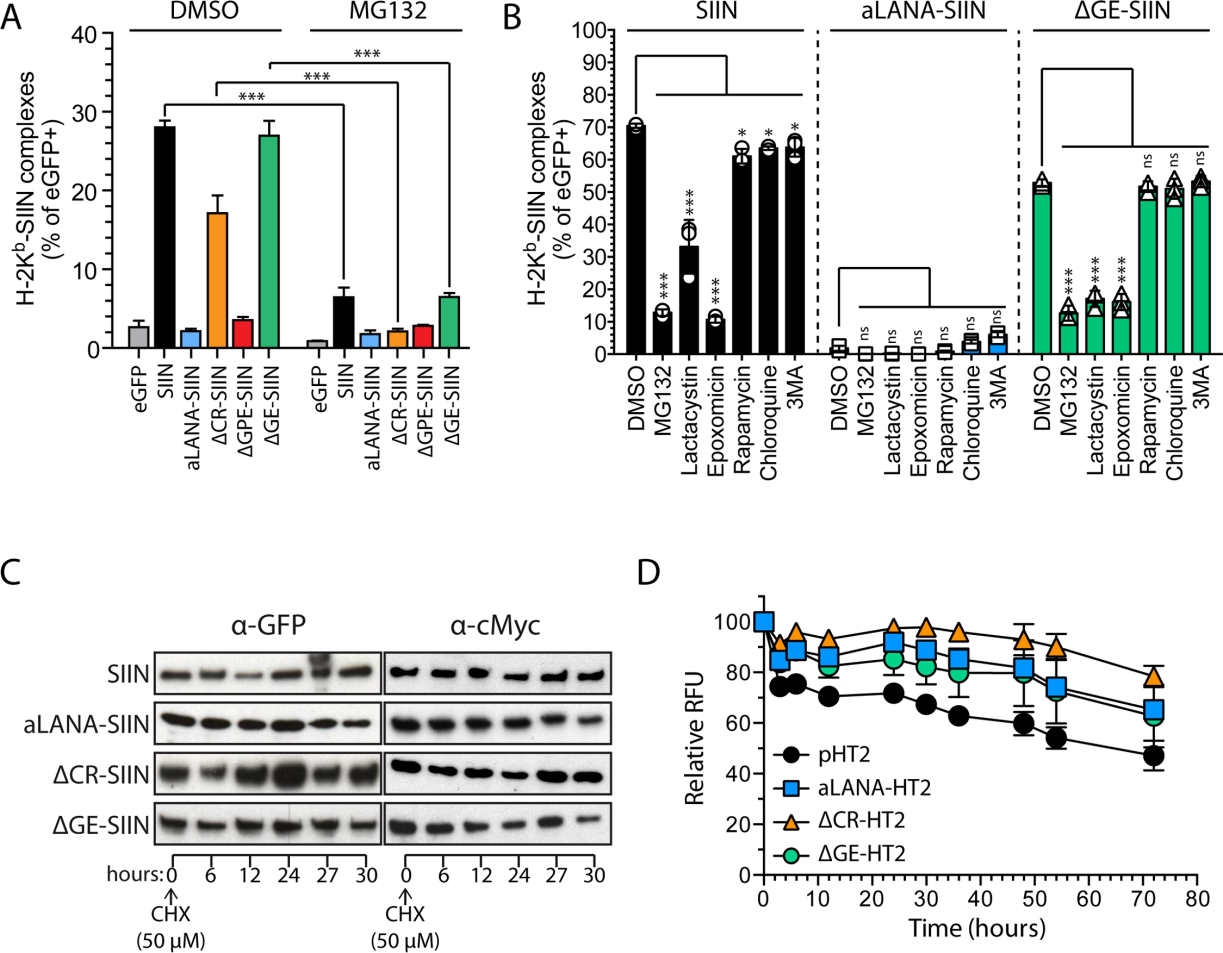
Rescued antigen presentation of OVA-peptide in absence of GE-rich domain is dependent on proteasomal degradation and absence of GE does not destabilize aLANA. (A) 293Kb cells were transfected with the different indicated constructs and treated 48h later with the proteasome inhibitor MG132 (20 μM) or vehicle (DMSO) for 16h before analysis by flow cytometry of H-2K^b^-SIINFEKL complexes expression at the cell surface. (B) Flow cytometry analysis of H-2K^b^-SIINFEKL expression in eGFP^+^ 293Kb cells 24h after transfection with SIIN, aLANA-SIIN or ΔGE-SIIN expression plasmids and treated during 16h with MG132 (20 μM), lactacystin (10 μM), epoxomicin (2 μM), rapamycin (20 μM), chloroquine (80 μM) or 3MA (10 mM). (C) Protein stability upon CHX-chase. 293Kb cells transfected with SIIN, aLANA-, ΔCR-, or ΔGE-SIIN expression plasmids were treated with CHX (50 μM) 24h post-transfection. Protein amounts were determined by Western blot analysis using anti-GFP antibody. (D) Protein stability upon HaloTag pulse-chase. 293Kb cells were transfected with the different indicated HaloTag (HT2) constructs during 48h, then pulsed with HaloTag TMR-Direct Ligand overnight, extensively washed and chased for 72h. Fluorometry analysis was performed at the indicated time points. Bars show mean ± S.D. (n = 3). Statistical analyses by one- (A) or two-way (B,D) ANOVA and Tukey’s (A) or Dunnett’s post-test with DMSO (B) or aLANA-HT (D) as control comparison mean (*p≤0.05, ***p≤0.001).

In order to decipher how GE inhibits *cis*-peptide presentation, we first examined the effect of proteasome inhibition on antigen presentation (Fig 6A). 293Kb cells were transfected to express eGFP, SIIN, aLANA, ΔCR-SIIN, ΔGPE-SIIN or ΔGE-SIIN before treatment with the proteasome inhibitor MG132 and detection of H-2Kb-SIINFEKL complexes at the cell surface by flow cytometry. MG132 treatment resulted in a severe reduction of antigen presentation for all constructs. Although MG132 mainly targets the proteasome, it can also affect autophagy pathways [37]. Thus, treatments using more specific lactacystin or epoxomicin were performed and resulted in significantly reduced detection of the H-2Kb-SIINFEKL complexes (Fig 6B and S3 Fig). However, treatments with rapamycin, chloroquine or 3 methyladenine (3-MA) did not affect antigen presentation. These results confirmed that GE-dependent inhibition of antigen presentation is dependent upon proteasomal degradation, whereas activation or inhibition of autophagy pathways did not affect peptide presentation. Inhibition of γ-HV GMPs epitope presentation by their respective central repeats has been attributed to reduced protein synthesis and reduced protein degradation [22]. To analyze the implication of the CR and GE regions in protein turnover, we first used a cycloheximide (CHX)/chase experiment at 24h after transfection (Fig 6C) and observed that the levels of proteins up to 30h after CHX treatment were not significantly affected in absence of CR or GE. In an alternative approach, we used the HaloTag technology to generate fusion proteins of aLANA, ΔCR and ΔGE. 293Kb cells were transfected with aLANA-, ΔCR- or ΔGE-HaloTag constructs and pulse-labeled overnight with the HaloTag TMR-Direct ligand. The cells were then washed and chased for 72h and fluorescence intensities were recorded over time (Fig 6D). All constructs showed similar turnover suggesting that the CR or GE regions are not involved in reduced protein degradation.

### The aLANA GE-rich domain regulates protein translation and RNA transcription levels

**Fig 7:**
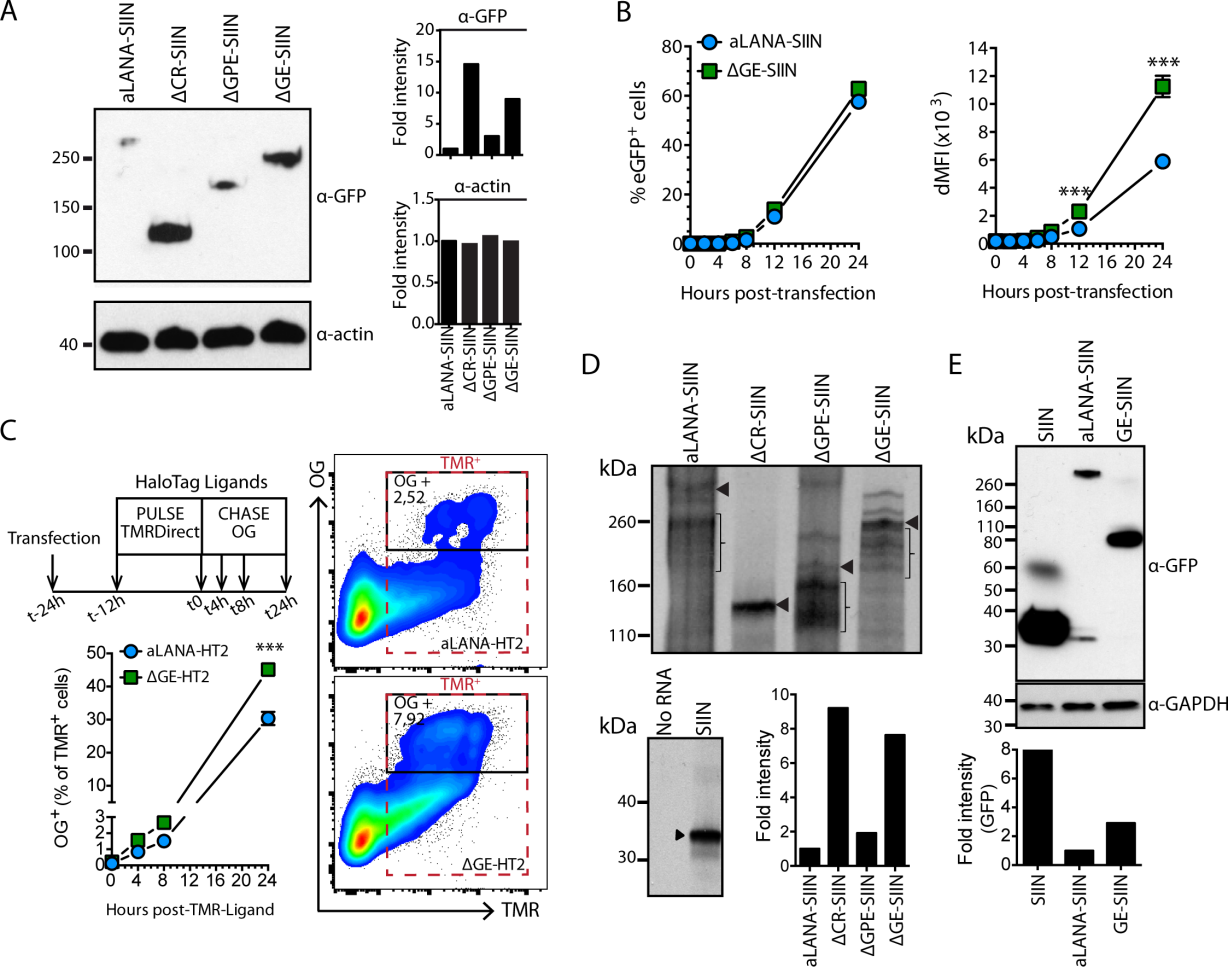
The GE-rich domain reduces protein synthesis efficiency of aLANA. (A) Immunoblot analysis of expression of aLANA-, ΔCR-, ΔGPE- and ΔGE-SIIN. 293Kb cells were transfected with the indicated plasmids and immunoblot performed at 48h after transfection. Duplicate blots were probed with either HRP-anti-GFP monoclonal antibody to detect aLANA constructs or anti-actin monoclonal antibody as loading control. Band intensities from the immunoblot were quantified by densitometry analysis using ImageJ64 software. Data are representative of three independent experiments with similar results. (B) Flow cytometry analysis of eGFP median fluorescence intensities (MFI) at the indicated time points after transfection. The left panel shows percent of eGFP^+^ cells and the right panel shows deltaMFI (dMFI). dMFI were calcutated as [MFI of eGFP-positive population–MFI of eGFP-negative population]. (C) Halotag pulse-chase. Cells were transfected with aLANA- or ΔGE-HT2 constructs and pulsed 12h later with HaloTag TMR-Direct Ligand overnight, extensively washed and chased for 24h. At the indicated time points, cells were incubated with cell-permeable HaloTag Oregon Green (OG) Ligand for 15 min before analysis by flow cytometry for detection of *de novo* protein expression. (D) Uncoupled *in vitro* transcription/translation assay of aLANA, ΔCR-, ΔGPE- and ΔGE-SIIN. The T7 promoter was first subcloned into the different peGFP-C1 constructs downstream the CMV promoter. The constructs were then transcribed *in vitro* with T7 RNA polymerase. Equimolar amounts of the resulting capped RNAs were then subjected to *in vitro* translation using rabbit reticulocyte lysate supplemented with [^35^S]-methionine. Intensities of the bands at the expected size obtained from immunoblotting in A were quantified by densitometry analysis using ImageJ64 software. Braces indicate radioactive signal from parasite bands. (E) Immunoblot analysis of SIIN, aLANA-SIIN, and GE-SIIN expression. 293Kb cells were transfected with the indicated plasmids and immunoblot performed at 24h after transfection. Duplicate blots were probed with either HRP-anti-GFP monoclonal antibody to detect aLANA constructs or anti-GAPDH monoclonal antibody as loading control. Band intensities from the immunoblot were quantified by densitometry analysis using ImageJ64 software. Data are representative of two independent experiments with similar results. Statistical analyses by two-way ANOVA and Sidak’s post-test (***p≤0.001).

Steady-state levels of protein expression were first investigated using immunoblotting of total cell lysates 48h after transfection with plasmids encoding aLANA-, ΔCR-, ΔGPE- or ΔGE-SIIN (Fig 7A). We observed that the absence of both CR and GE regions resulted in increased protein expression levels. In addition, we observed increased eGFP fluorescence intensities over time in absence of GE and at similar transfection efficiencies (Fig 7B). Thus, GE might regulate the level of protein expression. A pulse-chase labeling experiment was conducted to further address this hypothesis (Fig 7C). At 12h post-transfection of aLANA-, or ΔGE-HaloTag constructs, cells were pulse-labeled overnight with the HaloTag TMR-Direct ligand to label all transfected cells and chased for a further 24h. Then, *de novo* synthesized proteins were labeled with the HaloTag Oregon-Green (OG) ligand. We observed increased proportions of OG-positive cells over time in cells expressing ΔGE-HaloTag, suggesting that GE regulates the efficiency of protein synthesis. Next, we sought to directly investigate translation efficiency of aLANA-, ΔCR-, ΔGPE- or ΔGE-SIIN using an uncoupled *in vitro* translation assay (Fig 7D). Molar equivalents of capped RNA obtained from *in vitro* transcription using T7 RNA polymerase were translated *in vitro* using a rabbit reticulocyte lysate system. Although multiple bands could be observed for some constructs (S4B Fig), analysis of the intensity of the bands at the expected sizes (S4A Fig) suggested lower translation levels of aLANA and ΔGPE, while ΔCR and ΔGE showed higher translation efficiencies. Translation of SIIN was used as positive control and the negative control consisted of no RNA. These results suggested that the GE domain downregulates aLANA translation efficiency. In addition, immunoblotting of total cell lysates after transfection with SIIN, aLANA-SIIN or GE-SIIN expression vectors demonstrated that the GE domain was self-sufficient to significantly decrease protein steady-state levels after transfection of 293Kb cells (Fig 7E).

**Fig 8:**
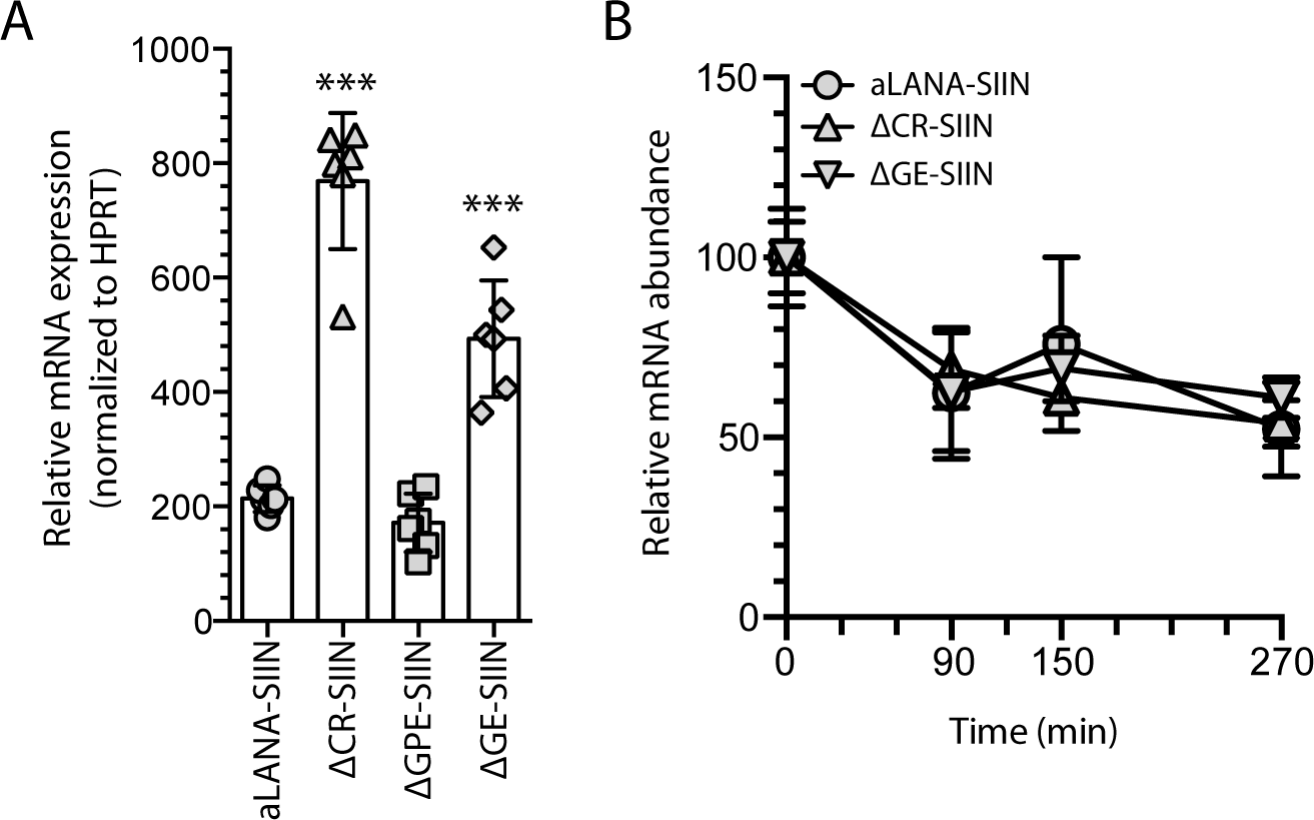
The GE-rich domain reduces mRNA transcription levels without affecting RNA stability. (A) Quantitative RT-PCR of aLANA-, ΔCR-, ΔGPE- and ΔGE-SIIN RNA extracted from 293Kb cells 48h after transfection with the different constructs and subjected to cDNA synthesis using oligo(dT) primers and quantitative PCR to detect ORF73 transcripts. (B) mRNA expression levels of aLANA-, ΔCR-, and ΔGE-SIIN after transcription inhibition. 293Kb cells were transfected with the indicated constructs and treated 48h later with actinomycin D to inhibit *de novo* transcription. Quantitative RT-PCR was then performed on total RNA extracted at the indicated time-points to detect ORF73 transcripts. Bars show mean ± S.D. (n = 3). Statistical analyses by one- (A) or two-way (B) ANOVA and Dunnett’s post-test with aLANA-SIIN as control comparison mean (***p≤0.001).

To further investigate whether the GE domain could also regulate transcription, we quantified steady-state RNA expression levels in 293Kb cells transfected with aLANA-, ΔCR-, ΔGPE- or ΔGE-SIIN. Interestingly, aLANA and ΔGPE transfected cells had similar low levels of specific transcripts whereas the absence of the CR or GE domains in aLANA resulted in significantly enhanced transcription levels (Fig 8A). Such increase of RNA expression was not due to decreased turnover of ORF73 mRNA as aLANA, ΔCR and ΔGE had similar RNA abundance after actinomycin D treatment following transfection (Fig 8B).

### Regulation to low-level protein expression by the GE-rich domain is associated with low presentation of linked antigenic peptide

**Fig 9:**
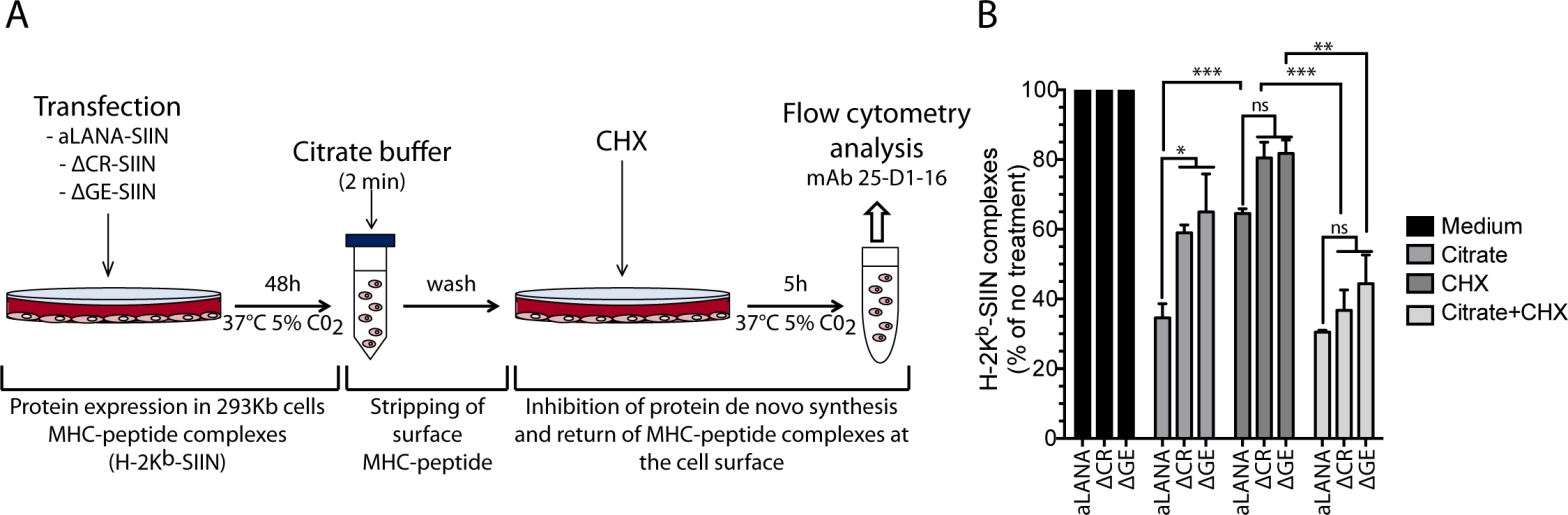
Effect of MHC-peptide stripping and cycloheximide treatment on antigen presentation. (A) 293Kb cells were transfected with aLANA-, ΔCR-, and ΔGE-SIIN constructs. At 24h post-transfection, cells were left untreated, treated with citrate phosphate buffer (pH 3), CHX (50μM), or treated with both citrate buffer and CHX. (B) The levels of H2-K^b^-SIINFEKL complexes expressed at the cell surface were then analyzed using an APC mouse anti-H2-K^b^-SIINFEKL complex (25-D1.16). Data represent the levels of H2-K^b^-SIINFEKL complexes relative to untreated cells. Bars show mean ± S.E.M (n = 3). Statistical analyses by two-way ANOVA and Tukey’s post-test (*p≤0.05, **p<0.01, ***p<0.001, ns, not significant).

The limited presentation efficiency of CD8^+^ T cell epitopes from EBNA1 has been suggested to be primarily determined by low translation efficiency rather than its intracellular stability [23, 29]. These observations were in line with the main hypothesis according to which defective ribosomal products (DRiPs) generated during protein synthesis rather than mature proteins are the major source of antigens that are processed to MHC class I [38–40]. The absence of the GE domain resulted in increased steady-state levels of aLANA protein expression that was related to increased translation efficiency and increased steady-state RNA levels (Figs 7 and 8), observations that could be associated with increased presentation of an endogenous epitope (Fig 4). To explore this possibility, we treated transfected 293Kb cells with citrate buffer to strip MHC-peptide from the cell surface, followed by incubation with CHX to inhibit *de novo* protein synthesis (Fig 9A). Combined treatments significantly reduced the proportion of cells expressing the MHC-peptide complexes at the cell surface irrespective of the presence or absence of repeat regions (Fig 9B). In contrast, CHX treatment alone had only a minimal effect on antigen presentation. Finally, citrate buffer treatment alone showed a differential effect on 293Kb expressing either aLANA or ΔCR and ΔGE, suggesting a more rapid antigen processing by MHC-I in absence of CR and GE. These results revealed an association between translation efficiency (and production of DRiPs) and antigen presentation.

### GE mRNA nucleotidic sequence regulates antigen presentation

**Fig 10:**
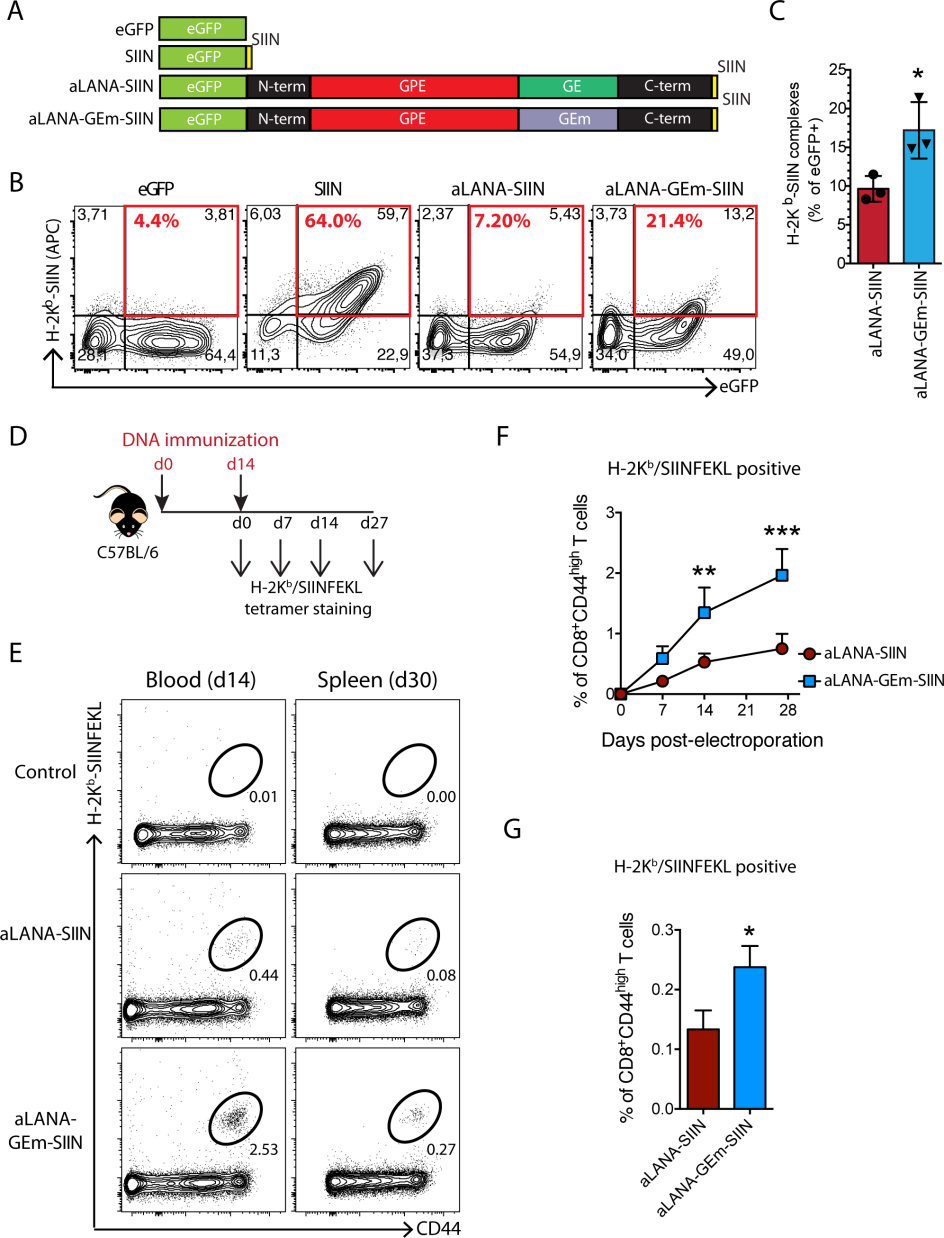
Codon-modified GE-rich domain of aLANA increases OVA-peptide presentation. (A) Schematic description of the aLANA expression constructs expressing either the native GE (aLANA-SIIN) or a codon-modified GE (aLANA-GEm-SIIN). (B) Representative results from flow cytometry analysis of SIINFEKL presentation on 293Kb cells. Cells were stained with APC mouse anti-H2-K^b^-SIINFEKL complex (25-D1.16) 48h after transfection with the indicated constructs. (C) Percent of eGFP^+^ cells expressing H2-K^b^-SIINFEKL complexes at the cell surface based on the analysis in B. Bars show mean ± S.D. (n = 3). (D) DNA immunization experimental layout. Mice received 20 μg of aLANA-SIIN or aLANA-GEm-SIIN plasmids in each tibial cranial muscle for electroporation. (E) Representative results from flow cytometry analysis of SIINFEKL-specific CD8^+^ T cells in peripheral blood at day 14 and in spleen at day 30 after the 2^nd^ immunization. Gated CD8^+^ T cells are shown. (F) Kinetics of SIINFEKL-specific CD8^+^ T cells in peripheral blood of immunized mice. (G) Percent of SIINFEKL-specific CD8^+^ T cells in spleen at day 30 after the 2^nd^ immunization. Statistical analyses by unpaired Student *t*-test (C and G, *p≤0.05) or two-way ANOVA and Sidak’s post-test (F, n = 4 to 6, *p≤0.05, **p≤0.01, ***p≤0.001).

Purine-rich GAr mRNA structure of EBNA1 regulates EBNA1 synthesis and presentation of EBNA1 to specific T cells [41–43]. Analysis of the nucleotidic sequence of the aLANA GE domain showed a bias towards usage of codons containing purines, with 100% of glutamate residues and 81% of glycine residues being purine codons (GAA, GAG and GGA) (S5A and S5B Fig) [27]. Prediction of mRNA secondary structure using Mfold revealed unstable secondary structures (S5C Fig). Thus, a codon-modified GE sequence (GEm) was synthesized in order to reduce purine bias, while conserving the identical peptidic sequence (S5A Fig). Modification of the codon usage resulted in increased stabilization of mRNA secondary structure reflected by the significantly more negative Gibbs free energy value (δG) of -153.91 kcal/mol compared with -43.39 kcal/mol for the native GE form (S5C Fig). We hypothesized that purine bias in aLANA GE mRNA sequence might be responsible for reduced antigen presentation. Pairwise mRNA sequence alignment of aLANA or aLANA-GEm with EBV EBNA1 demonstrated that the high sequence homology of aLANA GE with EBNA1 GAr sequence was lost after codon modification in GE (S5D Fig) [27]. Synthetic GEm sequence was used to replace the native GE in aLANA and generate the codon-modified aLANA-GEm (Fig 10A). Transfection of 293Kb cells aLANA-GEm-SIIN resulted in nuclear expression and significantly enhanced proportion of H2Kb-SIINFEKL expressing cells compared to native aLANA-SIIN (Fig 10B and 10C). Although the modification of the codon usage to reduce purine bias in GEm did not result in antigen presentation as high as observed in absence of GE (ΔGE-SIIN), our results suggest that constraints in native GE mRNA structure can limit antigen presentation. We further addressed this hypothesis by immunizing C57BL/6J mice using a DNA immunization protocol [21]. DNA immunization with GEm-SIIN expressing plasmid resulted in significantly increased SIIN-specific CD8^+^ T cells in peripheral blood compared to native aLANA-SIIN construct (Fig 10D–10G). These results demonstrated that mRNA constraints in GE limit antigen presentation and T cell priming.

### The absence of GE in aLANA does not impair MCF induction in rabbits

**Fig 11:**
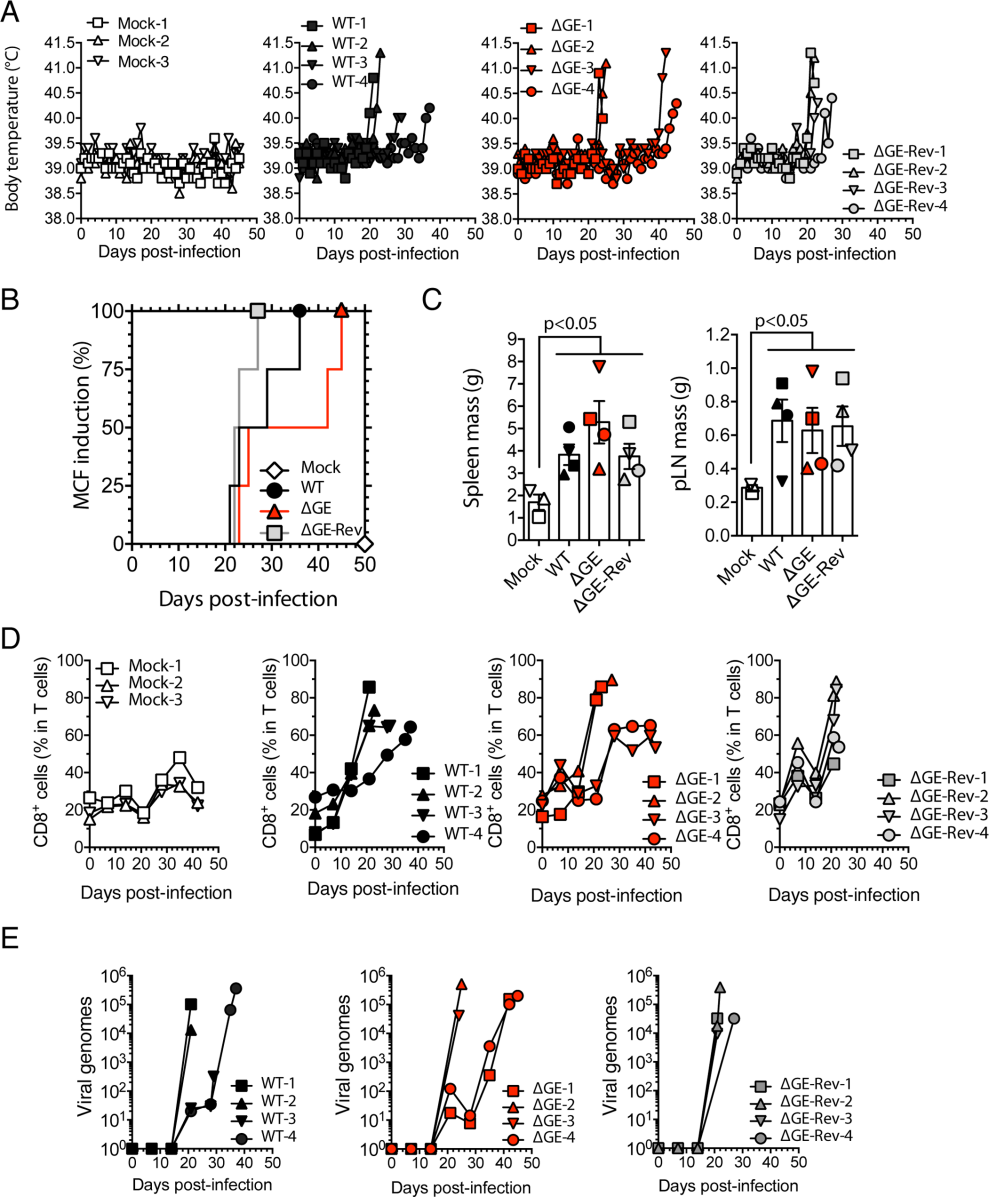
Malignant catarrhal fever induction. (A) Body temperature of 4 groups of rabbits infected intranasally with WT, ΔGE or ΔGE-Rev virus strains (10^5^ PFU/rabbit, n = 4). One group received the vehicle medium (Mock, n = 3). (B) Cumulative incidence of MCF induction in rabbits of the different groups (n = 4). (C) Tissue mass of spleen and popliteal lymph nodes (pLN) at the time of euthanasia. (D) Percentages of CD8^+^ cells in the gated T cell population analyzed by flow cytometry at regular intervals throughout the experiment. (E) Viral genome copy number in PBMC over time after infection. Bars represent mean ± SEM with symbols showing individual measurements. Statistical analyses by log-rank test (B) and one-way ANOVA and Dunnett’s post-test with Mock as control comparison mean (C).

We previously showed that MCF is caused by the lymphoproliferation of latently infected cells expressing high levels of aLANA [21]. Here, we observed that the absence of GE in aLANA resulted in increased presentation of an epitope linked to the protein (Fig 3). Based on previous reports showing that CTLs specific to EBNA1 or LANA1 could be detected in infected patients [6–8], that the EBNA1 GAr domain was dispensable for episome maintenance and immortalization of B cells *in vitro* [23], and that mLANA immune evasion of CTLs was essential for viral persistence in the host [34], we hypothesized that the absence of GE in aLANA might result in an enhanced CTL priming and impaired evasion of aLANA-specific CTLs. To address this hypothesis, we generated a recombinant virus expressing a GE-deleted (ΔGE) form of aLANA and its revertant (ΔGE-Rev) (S6A and S6B Fig). Viral growth in BT fibroblasts was not affected by the absence of aLANA GE domain (S6C Fig). This was expected as viruses impaired for aLANA expression had no growth defect [21]. We then infected rabbits intranasally with the WT, ΔGE or ΔGE-Rev strains (10^5^ PFU/rabbit). Rabbits infected with the ΔGE virus developed hyperthermia, splenomegaly, lymphadenopathy and expansion of CD8^+^ T cells similar to the WT and ΔGE-Rev control groups (Fig 11). Lymphoblastoid cell lines could also be propagated from both ΔGE and ΔGE-Rev-infected animals (S7 Fig), suggesting that a truncated form of aLANA lacking its GE region is able to maintain viral episomes. Together, these results suggest that aLANA GE-mediated inhibition of antigen presentation *in cis* is dispensable in MCF pathogenesis.

## Discussion

Wildebeest infection by AlHV-1 is persistent and nonpathogenic. Such an adaptation of the virus with its host species likely results from a long evolutionary relationship. Understanding the mechanisms acquired during coevolution by AlHV-1 to achieve such an adaptation in wildebeest and evade host immunity is important to develop strategies to target AlHV-1 latency in particular and γ-HV latent infection in general. In addition, AlHV-1 episomal persistence has been shown to be essential in MCF pathogenesis in susceptible species [21]. We have previously shown that a lack of aLANA expression rendered AlHV-1 unable to induce MCF, which positioned latency at the core of MCF pathogenesis. In this context, immune evasion by aLANA during MCF is likely of importance in the pathogenesis of this deadly disease. In the present study, we have brought evidence that aLANA can strongly limit antigen presentation and induction of antigen-specific cytotoxic T cell responses through a mechanism *in cis* depending on its GE-rich domain. However, AlHV-1 infection of MCF-susceptible rabbits induced MCF irrespective of the presence or absence of GE in aLANA.

We used transient expression of chimeric aLANA fusion proteins in a model of MHC class I peptide presentation in order to test the ability of AlHV-1 latency protein to evade immune detection. We observed a strongly reduced presentation of an epitope linked to aLANA. However, aLANA did not reduce antigen presentation *in trans* in co-transfection experiments. These results demonstrated that aLANA is not efficiently processed to MHC class I. Most viral maintenance proteins contain acidic repeat domains, such as for example the GAr domain of EBNA1, that have been involved in inhibition of MHC-I antigen presentation *in cis* [22]. aLANA has a long CR domain and antigen presentation could be restored after deletion of the entire CR sequence. We further investigated the role of subdomains within the CR region and identified a GE-rich region that was essential to inhibit the presentation of a linked peptide. Although subregions could be identified within the GE region, such as the GE1, E-rich and GE2 domains, only a protein lacking the GE-rich domain as a whole could restore antigen presentation and CTL activation to the levels of control chimeric eGFP-SIIN protein. In addition, we further observed that the transfer of GE to a heterologous eGFP-SIIN protein strongly inhibited antigen presentation. These results were important as they suggest that aLANA antigenic peptides are not effectively processed to MHC-I and identified the GE region as being sufficient to mediate immune evasion.

We observed that GE inhibits proteasome-dependent antigen presentation. Increased antigen presentation with ΔCR and ΔGE mutant proteins could have potentially been explained by increased susceptibility to proteasomal degradation. However, it was not the case as a lack of CR or GE did not result in increased protein turnover in pulse-chase experiments. Although the internal repeat region of several GMPs, including LANA1, EBNA1, and mLANA significantly decreased protein turnover [33, 34, 44], the protein stability of *saimiriine herpesvirus 2* (SaHV-2) ORF73 protein product was not influenced by the CR domain [31]. In addition, the region of LANA1 CR domain that inhibits proteasomal degradation was not involved in the inhibition of antigen presentation [32]. These data were supported by another study revealing that the half-life of a polypeptide did not determine antigen presentation [45], suggesting that protection from proteasomal degradation may not be sufficient to block antigen presentation.

Beside increased susceptibility to proteasomal degradation, several studies have shown that translation efficiency of GMPs such as EBNA1 is an important mechanism to explain the limited *cis*-antigen presentation [23, 24, 27–29]. Such observations resulted from a number of studies on EBNA1 over the last decade that showed that in spite of the inhibitory effect of the internal GAr domain, CTL responses directed towards EBNA1 could be readily detected in EBV seropositive individuals [7, 46, 47], and we could observe SIINFEKL-specific CTLs after DNA immunization with aLANA-SIIN (Fig 10). This paradox was resolved following extensive *in vitro* molecular analyses of the endogenous processing of EBNA1 indicating that CTL epitopes from this protein were predominantly generated from newly synthesized DRiPs rather than from the long-lived pool of stable EBNA1 in EBV-infected B cells [23, 26–28, 41, 47]. These observations have subsequently been further extended to demonstrate that the generation of DRiPs is intrinsically linked to the rate at which proteins are synthesized [23]. In the present study, we observed that the GE domain could reduce the efficiency of protein synthesis of aLANA, an observation that could be explained by a combination of increased translation efficiency (using uncoupled *in vitro* translation) as well as increased steady-state RNA expression levels when aLANA is deleted of GE. These results suggested that the GE-rich domain inhibits antigen presentation through regulation of both protein translation and RNA transcription levels, leading to a reduced production of DRiPs. DRiPs have been suggested to be the main source of viral antigens processed to MHC-I [48]. We therefore further hypothesized that targeting the mechanism leading to DRiPs production could be valuable for aLANA to ensure episome persistence during latency while avoiding detection from the immune system. Using citrate buffer and CHX co-treatment to block *de novo* protein synthesis after MHC-peptide stripping, we observed a significant reduction of detectable MHC-peptide complexes at the cell surface irrespective of the presence or absence of repeat regions. Thus, we can suggest that endogenous processing of CTL epitopes fused to the aLANA-ΔGE protein is not determined by its intracellular stability but rather by the rate at which newly synthesized polypeptides are produced.

Comparison of γ-HVs maintenance protein mRNA sequences revealed the presence of highly homologous purine-rich central repeat sequences [27]. Although highly conserved in mRNA sequence, these repeat regions encoded very different peptide sequences in the different viruses. In EBNA1, frame-shifting of the repetitive region without modifying the functional N- and C-terminal region did not significantly modify the reduced self-synthesis and the associated limitation *in cis* of antigen presentation. The purine-rich mRNA sequence would therefore be responsible for the reduced expression of these viral mRNAs rather than its encoded protein sequence. Interestingly, whereas the GE region of aLANA shares between 29.5 and 50% peptidic sequence homology with EBNA1 GAr region, the nucleotidic mRNA sequence corresponding to the GE region has more than 70% sequence homology with GAr and aLANA GE contains 96.6% purines. Codon modification of EBNA1 GAr sequence in order to reduce the purine-bias led to increased protein translation and improved CTL priming [26, 28]. We hypothesized that reducing the purine bias in the nucleotidic sequence of the GE region in aLANA would result in a better presentation of the epitope linked to it. Whereas both native aLANA and aLANA-GEm encode exactly the same protein, codon modification in GEm mRNA resulted in a significant increase of antigen presentation. Although the increased antigen presentation observed *in vitro* did not reach the levels obtained with a construct lacking GE, immunization of C57BL/6 mice with aLANA-GEm lead to the activation of significantly more antigen-specific CTLs (Fig 10). Inhibitory G-quadruplex structures were identified within EBNA1 nucleotidic sequence and are suggested to act as steric blocks to cause a stalling/dissociation of ribosomes, therefore reducing translation efficiency. Although G-quadruplex structures could also be predicted within aLANA central repeat [43], it is possible that the modification of the codon usage within GE was not sufficient to completely disrupt G-quadruplexes function for effective antigen presentation *in vitro* but sufficient to significantly increase cross-priming of specific CTLs *in vivo*. Further analyses are required to determine the presence of such structures in GE and their potential role in the *cis-*acting immune evasion of aLANA.

A rapid priming of an effective adaptive cellular immune response can determine the outcome of primary infection and also the control of persistent viral infections [49]. Infection of rabbits with a virus strain expressing a truncated form of aLANA lacking GE did not impair MCF induction. We previously showed that aLANA expression was essential for viral persistence and episomal maintenance during MCF. The present results suggest that the absence of GE did not impair episomal maintenance, which was further supported by the effective propagation of lymphoblastoid cells from rabbits infected by the ΔGE and ΔGE-Rev virus. The absence of GE did not render the protein unstable *in vitro* (Fig 7). We could therefore expect enhanced antigen-presentation by infected cells after infection with the ΔGE virus. Even though such enhanced anti-aLANA specific response could exist, it is not sufficient to clear proliferating infected cells and block the development of MCF lesions. A possible explanation would be the expression during MCF of viral proteins involved in the inhibition of antigen presentation by MHC-I *in trans*, though such proteins are yet to be identified in AlHV-1. This observation is important in our understanding of other lymphoproliferative diseases induced by γ-HV latent infection, where evasion mechanisms *in trans* could also be involved. However, our lack of knowledge of the proteins involved in such mechanisms in AlHV-1 infection renders this hypothesis currently difficult to address. Even though a *trans-*acting mechanism is possible, we have however previously shown that apart from aLANA-expressing ORF73, very scarce gene expression could be detected during MCF [19], which could suggest alternative immune evasion mechanisms developed by aLANA. Interestingly, EBNA1 was shown to be protected from proteasomal degradation through a mechanism depending on its high avidity for cellular DNA through the bipartite Gly-Arg repeat domains [50]. Potentially, similar domains outside GE could similarly protect aLANA against proteasomal degradation and therefore enable immune evasion *in vivo*. In addition, during KSHV and EBV lytic replication, alternative cytoplasmic isoforms of LANA1 and EBNA1 are expressed [51, 52]. Interestingly, a LANA1 isoform was shown to antagonize the innate immune DNA sensor cGAS [53]. Whether AlHV-1 also encodes such isoforms of aLANA and whether they can inhibit innate immunity is unknown. Nonetheless, their expression during lytic replication could significantly impair effective priming of aLANA-specific CD8^+^ T cells, thus potentially explain how a ΔGE virus is able to persist in an immunocompetent host and induce MCF.

In this study, we provide evidence that aLANA has evolved mechanisms to evade CTLs by inhibiting its own presentation in MHC-I through its central repeat purine-rich GE region that self-regulates protein synthesis and antigen processing to MHC class I. Importantly, our findings identify a key mechanism to evade immune surveillance by CTLs during AlHV-1 latency.

## Methods

### Ethics statement

The experiments, maintenance and care of mice and rabbits complied with the guidelines of the European Convention for the Protection of Vertebrate Animals used for Experimental and other Scientific Purposes (CETS n° 123). The protocol was approved by the Committee on the Ethics of Animal Experiments of the University of Liège, Belgium (Permits #1127, 1571 and 1598). All efforts were made to minimize suffering.

### Cell lines and virus strains

293A cells stably expressing murine class I alleles H-2K^b^ or H-2D^b^ (referred to as 293Kb and 293Db) were kindly provided by Dr Jonathan Yewdell (NIH/NIAID, USA) [36]. B3Z cells, an H2-K^b^-restricted CD8^+^ T-cell hybridoma, specific for the SIINFEKL ovalbumin peptide (OVA_254-264_/Kb) and L929-Kb cells were obtained from Dr P.G. Stevenson (University of Queensland, Australia). Bovine turbinate fibroblasts (BT, ATCC Crl-1390), bovine mammary epithelial cells stably expressing the NLS-Cre (MacT-Cre, ATCC CRL-10274) [54], Vero (ATCC CCL-81) and L929 (ATCC CCL-1) cells were cultured in Dulbecco’s modified essential medium (DMEM, Sigma) containing 10% FCS. Madin Darby bovine kidney cells (MDBK, ATCC CCL-22) were cultured in minimum essential medium (MEM, Sigma) supplemented with 10% FCS. The pathogenic AlHV-1 C500 strain isolated from an ox with MCF (initially obtained from Prof. D. Haig, University of Nottingham) and the AlHV-1 WT BAC clone were used in this study [18, 55]. Viruses were maintained by a limited number of passages (<5). Virus was amplified in BT cells before supernatants together with infected cells were concentrated by ultracentrifugation (100,000×g, 2h, 4°C) and suspended in PBS before storage at -80°C. Samples were thawed, clarified (100×g, 20 min, 4°C) and supernatants titrated by plaque assay as described previously [19].

### Plasmids

The peGFP-C1 (Clontech) expression vector was used to clone the chicken ovalbumin epitope SIINFEKL coding sequence into *Kpn*I/*Bam*HI sites using annealed oligonucleotides *Kpn*I/*Bam*HI-SIIN-Fwd and *Kpn*I/*Bam*HI-SIIN-Rev (S1 Table) and T4 DNA ligase (Roche) to generate the peGFP-SIIN vector. AlHV-1 ORF73 coding sequence was amplified by PCR using primers *Hin*dIII-73N-Fwd and *Kpn*I-73CΔstop-Rev (S1 Table) and AlHV-1 BAC DNA as template [18]. The obtained amplicon was then cloned into pGEM-T Easy vector (Promega) and subcloned by ligation into peGFP-SIIN to generate the peGFP-aLANA-SIIN plasmid. Subsequent plasmid constructs, including those containing the ORF73 coding sequence deleted of different subregions were generated either by ligation (T4 DNA ligase, Promega) or by homologous recombination using the In-Fusion HD cloning kit (Clontech) and primers listed in S1 Table. Plasmid vector peGFP-SIIN-aLANA was produced from peGFP-aLANA-SIIN after replacement of the SIINFEKL peptide sequence by a stop codon and in-frame insertion of SIINFEKL peptide sequence in N-terminus of ORF73 sequence. Plasmid vectors pT7-eGFP-73Δx-SIIN were generated by sub-cloning the T7 promoter sequence downstream the CMV IE promoter in the *Nhe*I site (S1 Table). Plasmid vector pEGFP-GE-SIIN was generated by insertion of ORF73 GE-rich domain sequence into pEGFP-SIIN. An additional SIINFEKL peptide sequence was further inserted in N-terminus of the GE sequence to create peGFP-SIIN-GE-SIIN (S1 Table). Plasmid vector pEGFP-aLANA-GEm-SIIN was generated from pEGFP-ΔGE1-SIIN. A pMK-RQ plasmid containing the synthetic GEm sequence (obtained by codon-optimization– http://eu.idtdna.com/CodonOpt –and further empirically modified to enrich in pyrimidines, then synthesized by GeneArt, Life technologies) was digested (*Pst*I*/Kpn*I) followed by ligation into pEGFP-ΔGE1-SIIN. Then, the ORF73 N-terminus coding sequence was inserted into *Kpn*I restriction site of pEGFP-ΔGE1-SIIN (S1 Table). HaloTag pHT2 expression vector (Promega) was generously provided by Prof. Fransen (KULeuven, Belgium) and used to produce the p-aLANA-HT2, pΔCR-HT2 and pΔGE-HT2 plasmids. To express eGFP-SIIN, aLANA-SIIN and ΔGE-SIIN constructs under control of the promoter EF1-α, pEFIN3 expression vector was digested with *Xba*I and used for insertion of PCR amplicons generated using the pEGFP-C1-based constructs as templates and primers EGFP-pEFIN3-Fwd and SIIN-pEFIN3-Rev (S1 Table). Plasmid vector pEFIN3-Kb to generate the VeroKb stable cell line was generated by insertion of H-2Kb PCR amplicon from pcDNA3.1-H2Kb (gift from Dr. J. Yewdell) into the *Xba*I site of pEFin3. All construct sequences were verified before being used in transfection experiments. mK3 expression vector was generously obtained from Dr P.G. Stevenson (University of Queensland, Australia).

### Antigen presentation assays

293Kb, 293Db, VeroKb or L929-Kb cells (10^5^ cells/well in 24-well plates) were transfected with 0.25 μg of plasmid using Fugene HD (Promega). For co-transfection assays, cells were co-transfected with 0.25 μg of each plasmid DNA. MHC-I-peptide complexes were detected by immunostaining using allophycocyanin (APC)-conjugated anti-mouse H-2K^b^-SIINFEKL complex (mouse IgG1, clone 25-D1.16, eBioscience) for 30 min on ice. Cells were incubated and washed in FACS buffer (PBS containing 0.1% BSA and 0.09% NaN_3_) before acquisition. Where indicated, transfected cells were treated with MG132 (Z-Leu-Leu-Leu-al), lactacystin, epoxomycin (Sigma), rapamycin, chloroquine or 3MA (InvivoGen) during 16h before immunostaining. Antigen presentation was assayed using B3Z cells (10^5^ cells/well in 24-well plates) co-cultured during 16h at 37°C with 293Kb cells transfected 48h before. Total cells were then lysed at −80°C during 30min in lysis buffer (5mM MgCL_2_, 1% Nonidet P-40, 0.15mM Chlorophenol red-β-D-galactopyranoside (CPRG, Invitrogen) in PBS) to quantify *β*-galactosidase activity. Each cell lysate replicate was then divided in 2 wells of a 96-well flat-bottom plate and incubated at 37°C during 20h. *β*-galactosidase activity was measured using a spectrophotometric determination of absorbance at 595 nm using an iMark microplate reader (Biorad).

### Confocal microscopy analysis

293Kb cells (2.5x10^5^ cells/well in 12-well plates) were grown on poly-D-lysine coated glass coverslips and transfected with 1 μg of plasmid using Fugene HD (Promega). Transfected cells were stained 20 min at room temperature with an anti-mouse H-2K^b^-SIINFEKL complex (25-D1.16, eBioscience), washed and then incubated 1h at 37°C with secondary Alexa Fluor 568 goat anti-mouse IgG polyserum (Life technologies). Cells were then washed and fixed in 4% paraformaldehyde in PBS before being mounted in ProLong antifade reagent (Life technologies). Washing and incubation steps were done in PBS containing 10% FCS. Fluorescence was then visualized with a Leica confocal scanner (TCS) SP laser scanning microscope.

### Western blotting

293Kb cells (5×10^5^ cells/well in 6-well plates) were transfected with 2μg of plasmid and Fugene HD (Promega). Cells were lysed after 24h in 100μL RIPA buffer (25mM Tris•HCl pH 7.6, 150mM NaCl, 1% Nonidet P-40, 1% sodium deoxycholate, 0.1% SDS). Lysates were heat-denatured (95°C, 5 min) before SDS-PAGE in laemmli buffer (31.25 mM Tris-Hcl pH 6.8, 1% (w/v) SDS, 12.5% (w/v) glycerol, 0.005% (w/v) bromophenol blue, Biorad) containing 10% (v/v) β-mercaptoethanol. Proteins were separated by electrophoresis in Mini-PROTEAN TGX precast 4–15% resolving gels (Biorad) in Tris-glycin running buffer (25mM Tris-base, 192mM glycine, 0.1% w/v SDS) and transferred onto polyvinylidene difluoride membranes (PVDF, Thermo Fisher Scientific, 0.45 μm pore size). The membranes were blocked with 3% skimmed milk in PBS/0.1% Tween-20. For detection of eGFP fusion proteins, membranes were blotted with HRP-conjugated anti-GFP antibody (mouse IgG1 mAb, 5000× diluted, Miltenyi Biotec). Mouse anti-*β*-actin (Dako) and anti-GAPDH (Abcam, GA1R) monoclonal antibodies were used with secondary HRP-conjugated rabbit anti-mouse polyserum (Dako). Rabbit anti-c-Myc polyserum (Sigma) was used with HRP-conjugated goat anti-rabbit polyserum (Dako). Novex Sharp pre-stained protein standard (Thermo Fischer) was used to determine band sizes. Chemiluminescent reaction was performed with ECL substrate (GE Healthcare) and membranes exposed onto an X-ray film.

### RNA extraction, cDNA synthesis and quantitative PCR

293Kb cells (2.5x10^5^ cells/well in 12-well plates) were transfected with 1 μg of plasmid using Fugene HD (Promega). Total RNA was extracted 24h after transection using RNeasy Miniprep kit (Qiagen) with on-column DNase I treatment. First-strand cDNA was then synthesized using oligo-dT and Superscript III Reverse transcriptase system (Life technologies). Control lacking reverse transcriptase was performed for each RNA sample to monitor DNA contamination. Quantitative real-time PCR reactions (qPCR) were performed in iQ-SYBR green supermix using a CFX96 Touch Real-Time PCR Detection System (Bio-Rad). Standard curves were generated using specific PCR amplicons and the obtained copy numbers were normalized to hypoxanthine-guanine phosphoribosyltransferase (HPRT) housekeeping gene (S2 Table). Where indicated, transfected cells were treated with actinomycin D (Sigma) during 4.5h before RNA extraction.

### Uncoupled *in vitro* transcription/translation

Capped RNAs were transcribed *in vitro* from *Avr*II-linearized pT7-eGFP-73Δx-SIIN plasmids using T7 RNA polymerase and mMessage mMachine kit (Ambion). Next, molar equivalents were used for uncoupled *in vitro* translation using a rabbit reticulocyte lysate system (Promega) and [S^35^]-methionine (Perkin Elmer). Translation reaction products were separated by SDS-PAGE before the gel was dried and exposed to an Amersham Hyperfilm MP (GE Healthcare).

### HaloTag pulse-chase assays

Pulse-labelling of live cells expressing HaloTag-fusion proteins was performed overnight at 12h post-transfection using cell-permeable HaloTag TMR-Direct Ligand (5μM, Promega). Cells were then extensively washed with warm PBS and chased in phenol red-free standard growth medium for the indicated period. At various time points, the fluorescence signal was measured using an EnSpire multimode plate reader (Perkin Elmer). In some experiments, pulse-labeled cells were further incubated at various time points of the chase period with cell-permeable HaloTag Oregon Green Ligand (5μM, Promega) during 15min at 37°C followed by analysis by flow cytometry.

### Cell surface MHC-peptide stripping and inhibition of *de novo* protein synthesis

To strip MHC-epitope complexes from the surface of 293Kb cells, cells were detached 48h post-transfection, washed with PBS and suspended in citrate phosphate (pH 3) buffer (0.131 M citric acid, 0.066 M Na_2_HPO_4_) for 2 min on ice [56]. The suspension was then neutralized with a 100-fold dilution of growth medium, and cells were washed twice. Then, cells were suspended in growth medium and incubated in the presence or absence of 100μg/ml CHX for 5 h (Sigma). Antigen presentation was assessed by flow cytometry analysis using anti-mouse H-2K^b^-SIINFEKL complex monoclonal antibody.

### Virus mutagenesis

The AlHV-1 BAC clone was used to produce the recombinant plasmids using the galactokinase gene (*galk*) as selection marker in SW102 *E*.*coli* strain [21]. To generate the BAC ΔGE plasmid, a BAC ΔGE-galK plasmid was first produced by positive selection in presence of an amplicon obtained by PCR using the p*galK* vector as a template, the forward chimeric primer H1ΔGE-galK-Fwd and the reverse chimeric primer H2ΔCR-galK-Rev (S3 Table). The amplicon consisted in the *galK* sequence flanked by 44-bp sequences corresponding to the regions directly flanking the GE region of the AlHV-1 ORF73 (access no.: Refseq NC_002531, nt 117121–117170 and 117948–117991) in the viral genome. Full deletion of the GE region was further achieved by *galK* negative selection using annealed oligonucleotides H1H2-ΔGE-Fwd and H1H2-ΔGE-Rev. Finally, the BAC ΔGE-Rev plasmid was produced using two sequential steps consisting in the re-introduction of the *galK* sequence using the ΔGE-galK amplicon before negative selection performed in presence of the ORF73 full length fragment obtained by *Sac*I digestion of the pEGFP-aLANA-SIIN plasmid (S6 Fig). All deletions and insertions were further verified using restriction endonuclease and Southern blot approaches and sequencing of the recombination sites. All strains were reconstituted in MacT-Cre cells before propagation in BT cells.

### Southern blot

Southern blotting analysis was performed as described previously [19].

### Growth curves

*In vitro* growth kinetics of recombinant viruses were compared to those of the WT. Cells were infected (moi = 0.05) and both supernatants and infected cells were harvested at successive intervals. The total amount of infectious viral particles was determined by plaque assay on MDBK cells as described previously [19].

### MCF induction

Four groups of specific pathogen-free New-Zealand white rabbits (n = 4) were used. Animals were inoculated intranasally with 10^5^ PFU of the different AlHV-1 recombinant viruses in PBS or PBS only for the mock-infected group. Rabbits were examined daily for clinical signs. According to bioethical rules, rabbits were euthanized when rectal temperature remained higher than 40°C for two consecutive days.

### Leukocytic cell suspension preparation

PBMC were isolated from 5-ml of blood collected from the ear central artery before and at different time-points after infection. Immediately after euthanasia, single-cell suspensions were prepared from popliteal lymph node (pLN) and spleen as follows. Tissue biopsies were delicately chopped in sterile RPMI media and passed through a 70 μm cell-strainer (BD Biosciences). Mononuclear leukocyte suspensions from peripheral blood and tissue samples were prepared with Ficoll-Paque Premium density gradient media (GE Healthcare). 5-ml single-cell suspension was diluted 1:1 in sterile PBS, overlaid onto 5-ml Ficoll-Paque density cushion and centrifuged (1825×*g*) during 20-min at room temperature. Mononuclear leukocytes at the interface were collected and washed in ice-cold PBS before further analysis.

### DNA immunization

8-week-old female C57BL/6 mice were subjected to DNA immunization as described previously [21]. Briefly, 20 μg of plasmid construct in 25 μL of PBS were electroporated in both hind legs at day 0 and 14 in the tibial cranial muscle. Blood sampling were taken at d0, 7, 14 and 27 before euthanasia and spleen harvested at d30.

### Antibodies and flow cytometry

Multi-color flow cytometry analysis of rabbit PBMC was performed as described previously [20]. Briefly, cells were incubated in FACS buffers (PBS containing 0.1% BSA, 0.09% NaN_3_) with mAb anti-rabbit CD4 (IgG2a, KEN-4), CD8 (IgG1, 12C.7), IgM (IgG1, NRBM) antibody cocktail and left on ice for 10min. Cells were washed and further incubated for 10min on ice with isotype-specific PE-conjugated rat anti-mouse IgG1 (A85-1, BD) and biotinylated rat anti-mouse IgG2a (R19-15, BD) antibodies. After a third wash, cells were incubated with FITC-conjugated anti-rabbit T cells (KEN-5), and APC-conjugated streptavidin (BD) before washing, staining with 7-AAD and acquisition. All antibodies were from AbD-Serotec-Biorad or specified otherwise.

Following DNA immunization, blood samples were collected from mice at regular intervals, lysed in ACK lysis buffer (Gibco) following the manufacturer’s instructions and incubated in FACS buffer with mAb anti-mouse CD3 (APC, 145-2C11), CD8α (FITC, 53–6.7), CD44 (PE-Cy7, IM7). Antibodies were from BD Biosciences. Samples were further incubated with H-2K^b^-SIINFEKL tetramer (Brilliant violet 421nm, NIH Tetramer Core Facility).

### Flow cytometry

Acquisitions were performed using a LSR Fortessa X-20 (BD Biosciences). A total of 50 to 100,000 live events were collected and data were analyzed by Flowjo v10.0.7 software (Treestar).

### Statistical analysis

Statistical analyses were conducted using Graphpad Prism v6 software (GraphPad, San Diego, CA). Unpaired Student’s *t* test was conducted when comparing statistical difference between two datasets. One or two-way ANOVA were conducted when comparing more than two groups of data as indicated in the figure legends and followed by relevant post-test for mean multiple comparison. Sidak’s method was used when comparing pairs of means within each row, and Dunnett’s or Tukey’s methods were chosen for comparing every mean (3 or more columns) within each row to one control mean or every mean with every other mean, respectively.

## Supporting information

S1 Fig aLANA GE-mediated inhibition of antigen presentation. (A) 293Kb or 293Db cells were transfected with the indicated constructs and stained 24h later for the detection of H-2K^b^-SIINFEKL complexes on the cell surface. Numbers in bold indicate the percent of H-2K^b^-SIINFEKL-positive cells within eGFP^+^ cells (n = 3). (B) L929-Kb cells were transfected with eGFP, SIIN or aLANA-SIIN expression vectors and stained 48h later for detection of H-2K^b^-SIINFEKL-positive cells (n = 3 to 9). (C) VeroKb cells stably expressing the mouse H-2K^b^ haplotype were generated using the pEFIN3-Kb plasmid construct as detailed in the methods and selection was performed using geneticin (250 μg/mL). VeroKb cells were used without further cloning and transfected with the indicated constructs and stained 24h later for the detection of H-2K^b^-SIINFEKL complexes on the cell surface. Representative plots are shown and numbers in boxes indicate the percent of H-2K^b^-SIINFEKL-positive cells within eGFP^+^ cells. (D) 293Kb cells were transfected with pEFIN3-SIIN, -aLANA-SIIN, or -ΔGE-SIIN and analyzed 24h later for the expression of H-2K^b^-SIINFEKL complexes. Numbers in boxes indicate the percent of H-2K^b^-SIINFEKL-positive cells within eGFP^+^ cells. Bar graph show the percent of eGFP^+^ cells expressing H-2K^b^-SIINFEKL complexes. Statistical analyses by two-way (A) or one-way ANOVA (B,D) and Dunnett’s post-test with eGFP (A), SIIN (B) or aLANA-SIIN (D) as control comparison mean (**p≤0.01, ***p≤0.001).
(TIF)

S2 Fig Epifluorescence microscopic analysis of expression of fusion proteins in 293Kb cells 48h after transfection with the different constructs. Nuclear DNA is stained in blue using 4’,6-diamidino-2-phenylindole (DAPI).
(TIF)

S3 Fig Epoxomicin and lactacystin treatment. 293Kb cells were transfected and treated 24h later with the indicated concentrations of proteasome inhibitors during 16h. Cells were then stained to detect surface H-2K^b^-SIINFEKL complexes. The results show the percent of eGFP^+^ cells expressing H-2K^b^-SIINFEKL complexes.
(TIF)

S4 Fig Western blot and *in vitro* translation assay. (A) eGFP-tagged proteins were detected by western blot 24h after transfection. Loading of lysates was adapted in order to obtain detectable bands and determine the molecular weight of aLANA-, ΔCR-, ΔGPE- and ΔGE-SIIN proteins. (B) *In vitro* translation assay. Original gel used to generate Fig 7D and illustrating how the bands at the expected sizes were determined.
(TIF)

S5 Fig Codon-modification of the GE-rich region of aLANA. (A) Sequence alignment between the native GE and codon-modified GEm sequences. Matched and mismatched nucleotides are depicted in blue and red, respectively. (B) Codon usage of native GE (GE), codon-optimized GE obtained using CodonOpt (IDTDNA) (GEopt) and codon-modified GE (GEm) sequences. (C) Prediction of mRNA secondary structures using Mfold of GE, GEopt and GEm sequences. ΔG; Gibbs free energy value. (D) Dot-plot analysis illustrating pairwise local alignement between EBV EBNA1 mRNA sequence and the mRNA sequence of aLANA or aLANA-GEm. The overall homology is shown as a straight line on the diagonal, while regions of homologous repeats are shown as lots of lines in the same region. Both EBNA1 GAr and aLANA GE repeat mRNA sequences are identified using broken lines boxes. Alignements were performed using zPicture (https://zpicture.dcode.org), a dynamic alignment and visualization tool based on the BLASTZ alignment program.
(TIF)

S6 Fig Production of aLANA-ΔGE virus. (A) Schematic representation of the recombineering methodology used to produce the viral recombinant strains expressing truncated forms of aLANA lacking the GE domain (ΔGE). (B) The produced BAC plasmids were analyzed by Southern blotting after *Sac*II restriction and ethidium bromide staining (EtBr). ORF73 probe consisted in a 679-bp C-terminal region of ORF73 coding sequence. (C) Multi-step growth curves of WT, ΔGE, or ΔGE-rev virus strains in BT fibroblasts. The data presented are the means ± SD of results from measurements in triplicates.
(TIF)

S7 Fig Lymphoblastoid cell lines (LCLs) phenotypic analysis. LCLs were propagated from peripheral blood mononuclear cells of rabbits developing MCF after infection with ΔGE or ΔGE-rev viruses in Iscove’s modified Dulbecco’s medium (IMDM) containing 10% FCS and supplemented with recombinant human interleukin 2 (Roche, 10 IU/mL). Cells were maintained in medium replaced every 3–4 days and analyzed by flow cytometry for IgM^+^ B cells, CD8^+^ T cells and CD4^+^ T cells after 3 weeks culture (A). (B) Percent of CD8^+^ T cells in LCLs based on analysis in A. (C) LCL cell counts over time in culture.
(TIF)

S1 Table Oligonucleotides used in this study to produce aLANA and aLANA mutant expression plasmids. Restriction sites in the primers sequences were highlighted in bold.
(PDF)

S2 Table Oligonucleotides used in this study to perform quantitative PCR reactions. (PDF)

S3 Table Oligonucleotides used in this study to produce BAC recombinant expression plasmids. (PDF)
