# Supplementary figures and images for: Macavirus latency-associated protein evades immune detection through regulation of protein synthesis in cis depending upon its glycin/glutamate-rich domain
Source: PLoS Pathog. 2017 Oct 23;13(10):e1006691. doi: 10.1371/journal.ppat.1006691 (PMC5695634; doi:10.1371/journal.ppat.1006691)

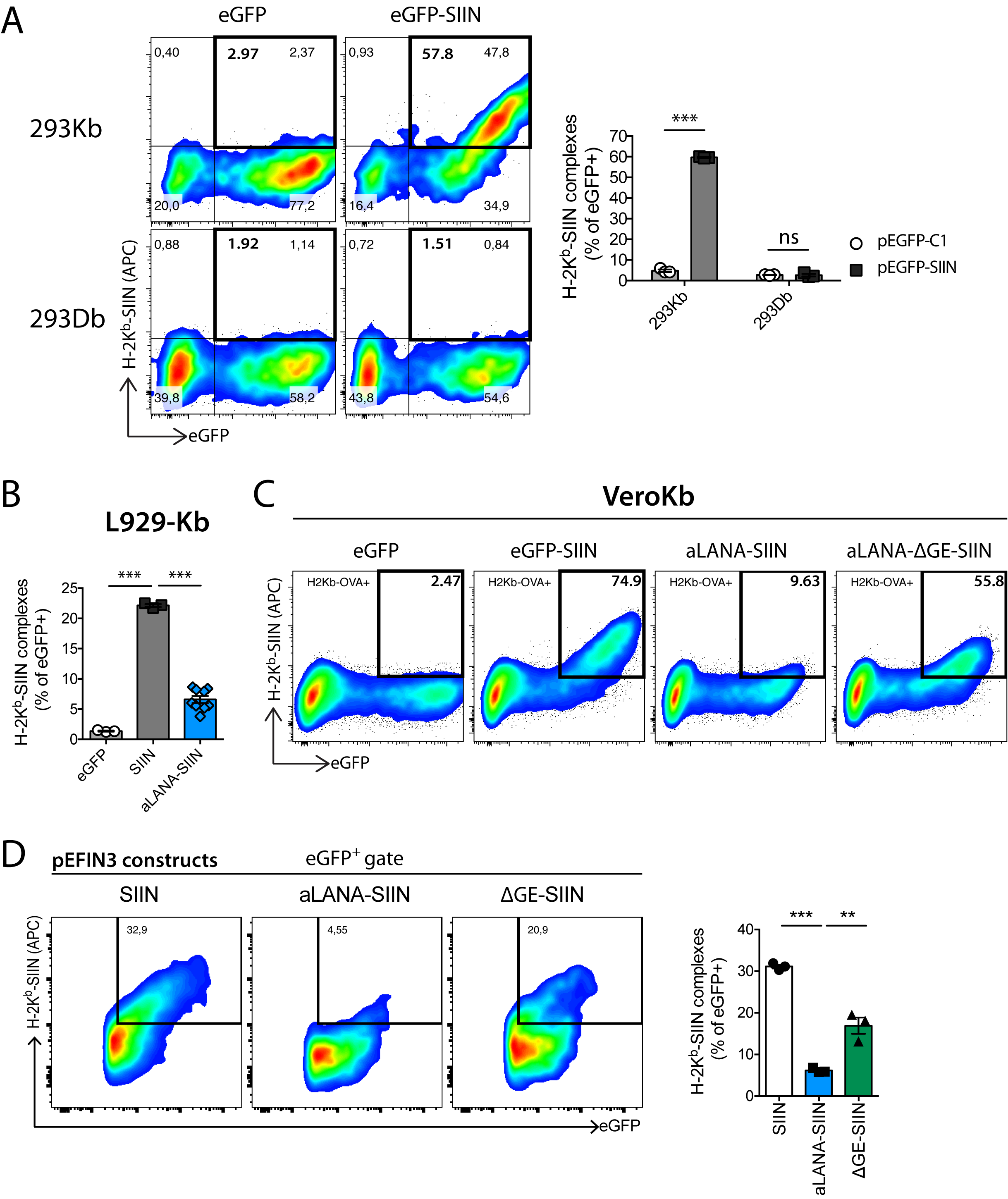

Supplement: S1 Fig — (A) 293Kb or 293Db cells were transfected with the indicated constructs and stained 24h later for the detection of H-2Kb-SIINFEKL complexes on the cell surface. Numbers in bold indicate the percent of H-2Kb-SIINFEKL-positive cells within eGFP+ cells (n = 3). (B) L929-Kb cells were transfected with eGFP, SIIN or aLANA-SIIN expression vectors and stained 48h later for detection of H-2Kb-SIINFEKL-positive cells (n = 3 to 9). (C) VeroKb cells stably expressing the mouse H-2Kb haplotype were generated using the pEFIN3-Kb plasmid construct as detailed in the methods and selection was performed using geneticin (250 μg/mL). VeroKb cells were used without further cloning and transfected with the indicated constructs and stained 24h later for the detection of H-2Kb-SIINFEKL complexes on the cell surface. Representative plots are shown and numbers in boxes indicate the percent of H-2Kb-SIINFEKL-positive cells within eGFP+ cells. (D) 293Kb cells were transfected with pEFIN3-SIIN, -aLANA-SIIN, or -ΔGE-SIIN and analyzed 24h later for the expression of H-2Kb-SIINFEKL complexes. Numbers in boxes indicate the percent of H-2Kb-SIINFEKL-positive cells within eGFP+ cells. Bar graph show the percent of eGFP+ cells expressing H-2Kb-SIINFEKL complexes. Statistical analyses by two-way (A) or one-way ANOVA (B,D) and Dunnett’s post-test with eGFP (A), SIIN (B) or aLANA-SIIN (D) as control comparison mean (**p≤0.01, ***p≤0.001). (TIF) [file ppat.1006691.s001.tif]

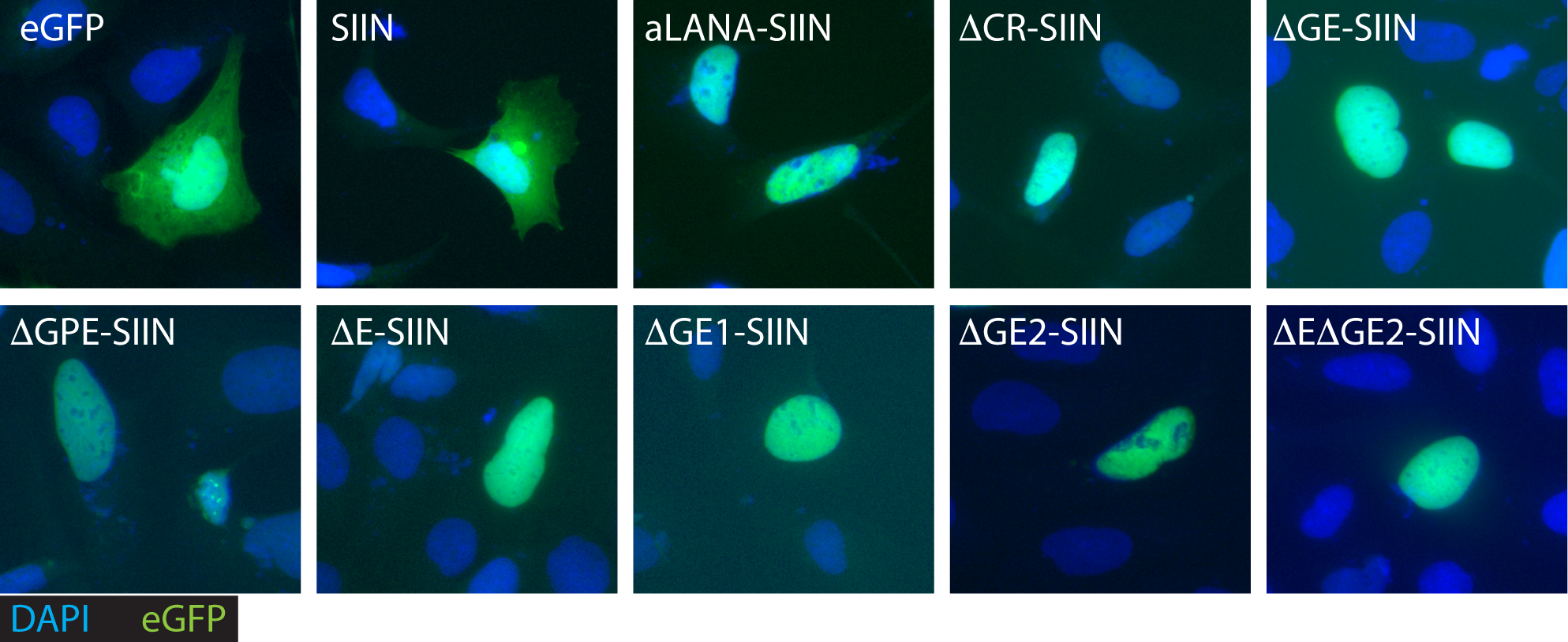

Supplement: S2 Fig — Nuclear DNA is stained in blue using 4’,6-diamidino-2-phenylindole (DAPI). (TIF) [file ppat.1006691.s002.tif]

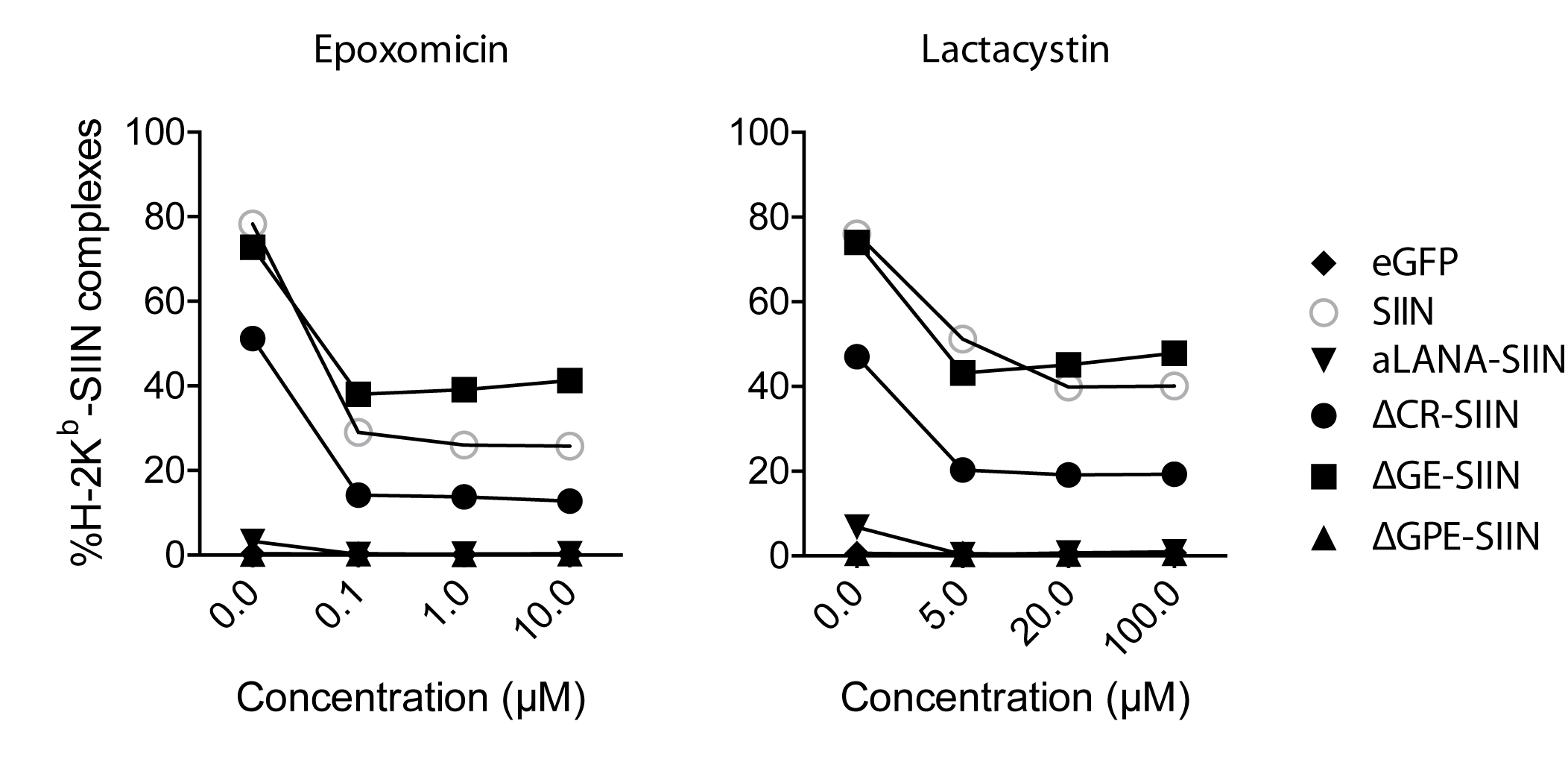

Supplement: S3 Fig — Cells were then stained to detect surface H-2Kb-SIINFEKL complexes. The results show the percent of eGFP+ cells expressing H-2Kb-SIINFEKL complexes. (TIF) [file ppat.1006691.s003.tif]

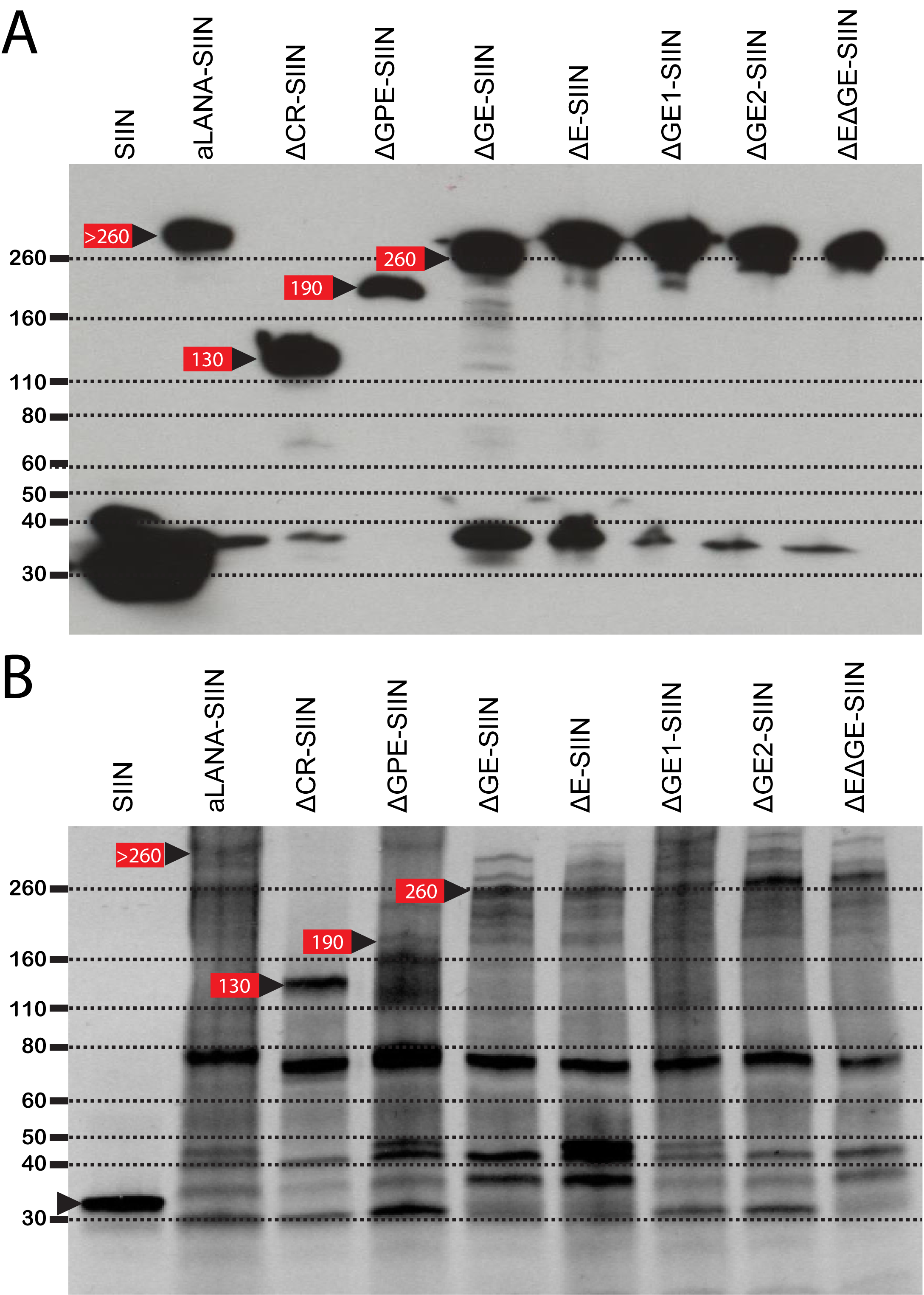

Supplement: S4 Fig — (A) eGFP-tagged proteins were detected by western blot 24h after transfection. Loading of lysates was adapted in order to obtain detectable bands and determine the molecular weight of aLANA-, ΔCR-, ΔGPE- and ΔGE-SIIN proteins. (B) In vitro translation assay. Original gel used to generate Fig 7D and illustrating how the bands at the expected sizes were determined. (TIF) [file ppat.1006691.s004.tif]

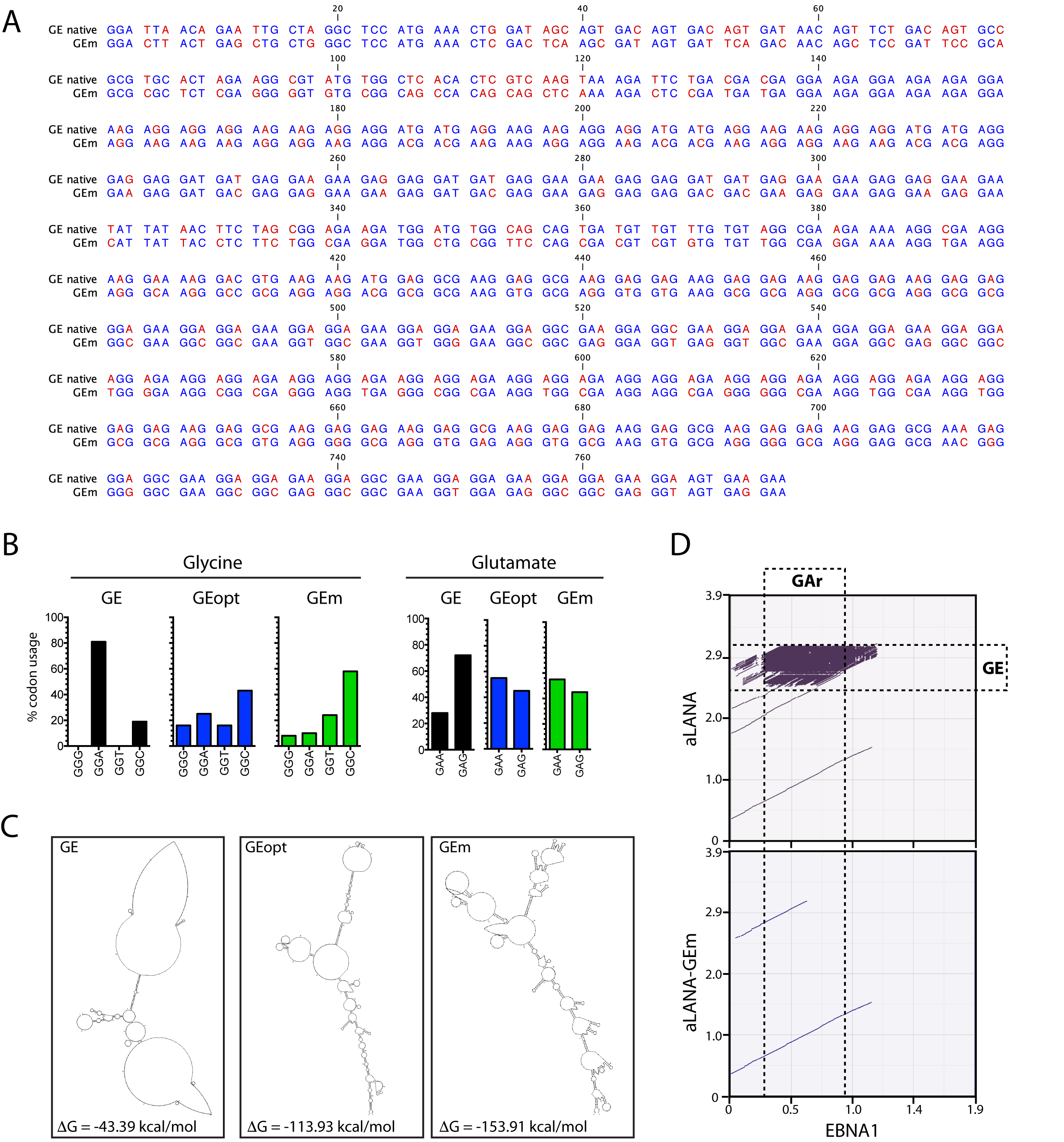

Supplement: S5 Fig — (A) Sequence alignment between the native GE and codon-modified GEm sequences. Matched and mismatched nucleotides are depicted in blue and red, respectively. (B) Codon usage of native GE (GE), codon-optimized GE obtained using CodonOpt (IDTDNA) (GEopt) and codon-modified GE (GEm) sequences. (C) Prediction of mRNA secondary structures using Mfold of GE, GEopt and GEm sequences. ΔG; Gibbs free energy value. (D) Dot-plot analysis illustrating pairwise local alignement between EBV EBNA1 mRNA sequence and the mRNA sequence of aLANA or aLANA-GEm. The overall homology is shown as a straight line on the diagonal, while regions of homologous repeats are shown as lots of lines in the same region. Both EBNA1 GAr and aLANA GE repeat mRNA sequences are identified using broken lines boxes. Alignements were performed using zPicture (https://zpicture.dcode.org), a dynamic alignment and visualization tool based on the BLASTZ alignment program. (TIF) [file ppat.1006691.s005.tif]

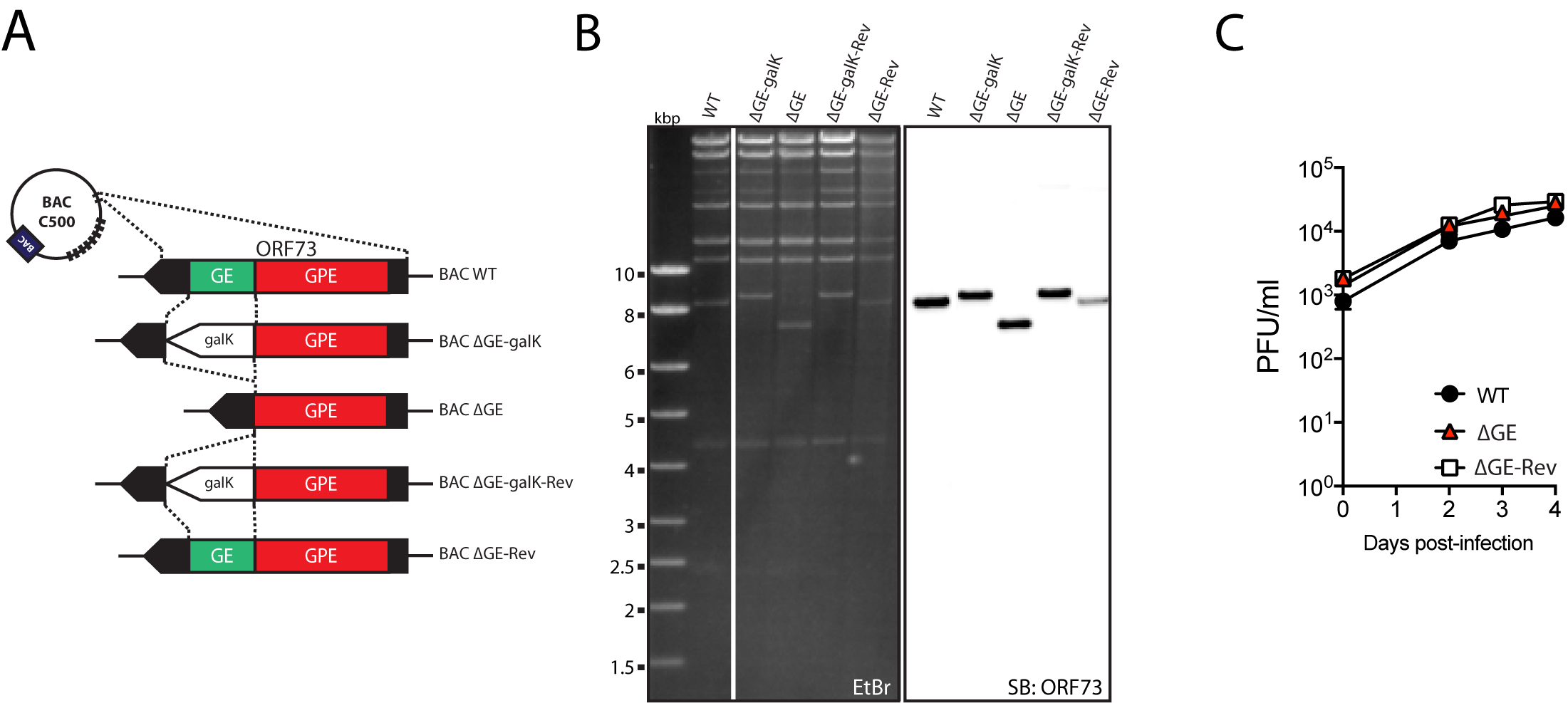

Supplement: S6 Fig — (A) Schematic representation of the recombineering methodology used to produce the viral recombinant strains expressing truncated forms of aLANA lacking the GE domain (ΔGE). (B) The produced BAC plasmids were analyzed by Southern blotting after SacII restriction and ethidium bromide staining (EtBr). ORF73 probe consisted in a 679-bp C-terminal region of ORF73 coding sequence. (C) Multi-step growth curves of WT, ΔGE, or ΔGE-rev virus strains in BT fibroblasts. The data presented are the means ± SD of results from measurements in triplicates. (TIF) [file ppat.1006691.s006.tif]

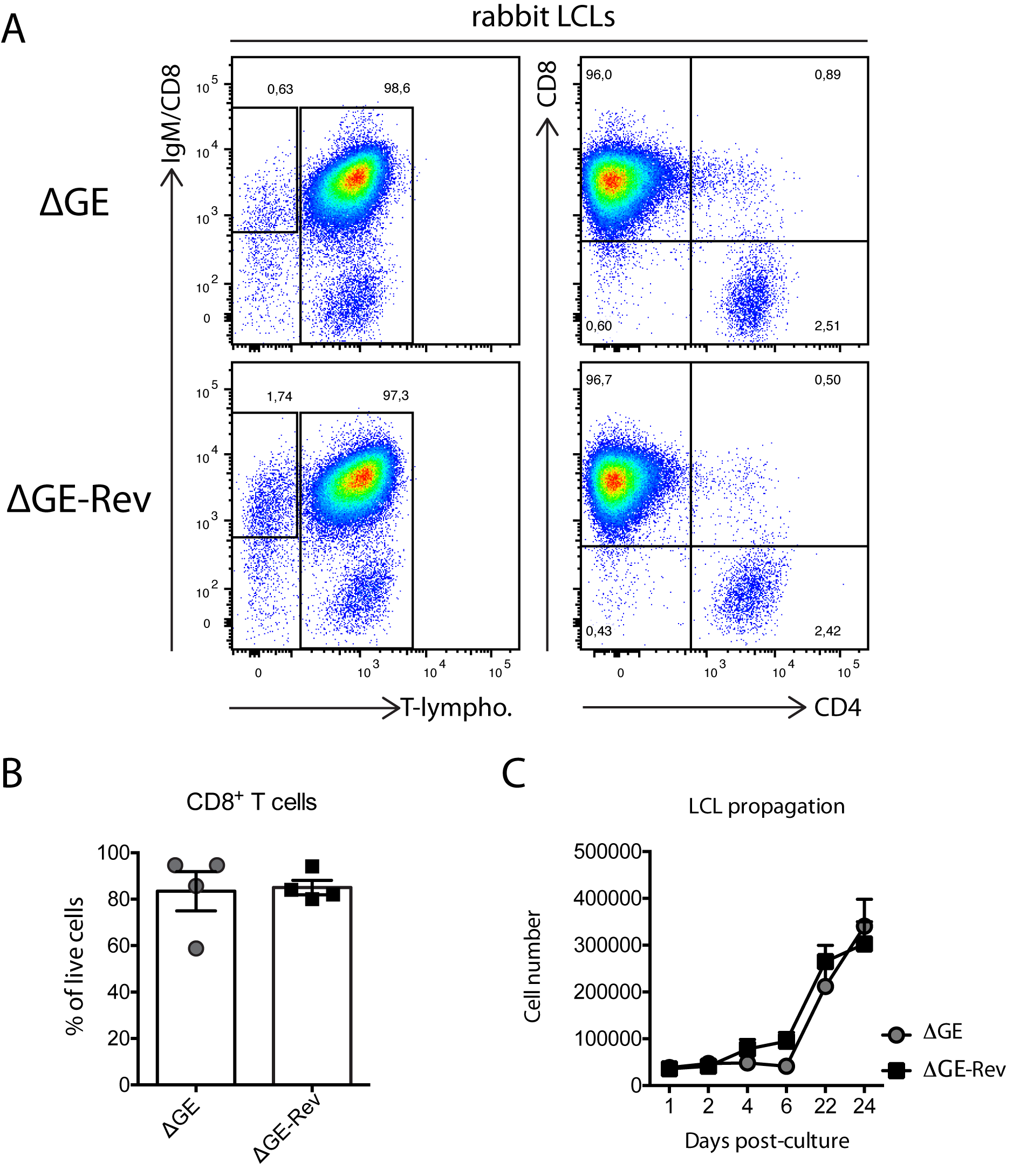

Supplement: S7 Fig — LCLs were propagated from peripheral blood mononuclear cells of rabbits developing MCF after infection with ΔGE or ΔGE-rev viruses in Iscove’s modified Dulbecco’s medium (IMDM) containing 10% FCS and supplemented with recombinant human interleukin 2 (Roche, 10 IU/mL). Cells were maintained in medium replaced every 3–4 days and analyzed by flow cytometry for IgM+ B cells, CD8+ T cells and CD4+ T cells after 3 weeks culture (A). (B) Percent of CD8+ T cells in LCLs based on analysis in A. (C) LCL cell counts over time in culture. (TIF) [file ppat.1006691.s007.tif]
