# Supplementary material for: Macavirus latency-associated protein evades immune detection through regulation of protein synthesis in cis depending upon its glycin/glutamate-rich domain
Source: PLoS Pathog. 2017 Oct 23;13(10):e1006691. doi: 10.1371/journal.ppat.1006691 (PMC5695634; doi:10.1371/journal.ppat.1006691)
Supplement: S1 Table — Restriction sites in the primers sequences were highlighted in bold. (PDF) [file ppat.1006691.s008.pdf]

S1 Table. Oligonucleotides used in this study to produce aLANA and aLANA mutant expression plasmids

Construction	Primer	Sequence	Strategy
**SIIN**	*Kpn*I/*Bam*HI-SIIN-Fwd	5'-**ggtacc**agcataattaatttcgaaaagctctaagcggccgcg**ggatcc**-3'	**Ligation**
	*Kpn*I/*Bam*HI-SIIN-Rev	5'-**ggatcc**cgcggccgcttagagcttttcgaaattaattatgct**ggtacc**-3'	
**aLANA-SIIN**	*Hind*III-73N-Fwd	5'-**aagctt**ggatggtgcttctaagatcagg-3'	**Ligation**
	*Kpn*I-73CΔstop-Rev	5'-**ggtacc**ttcatgtggaaatggaaagg-3'	
**SIIN-aLANA**	73-*Kpn*I-TAA-*Bam*HI-Fwd	5'-ccatttccacatgaa**ggtacc**taacccggg**ggatcc**accggatctagataa-3'	**Recombination**
	73-*Kpn*I-TAA-*Bam*HI-Rev	5'-ttatctagatccggt**ggatcc**cccgggtta**ggtacc**ttcatgtggaaatgg-3'	
	H-SIIN-*H*-73FL-Fwd	5'-tcagatctcgagctc**aagctt**ggcccgggagcataattaatttcga<br>aaagctgg**aagctt**ggatggtgcttctaa-3'	
	H-SIIN-*H*-73FL-Rev	5'-ttagaagcaccatcc**aagctt**ccagcttttcgaaattaattatgctccc<br>gggcc**aagctt**gagctcgagatctga-3'	
**ΔCR-SIIN**	*Hind*III-73N-Fwd	5'-**aagctt**ggatggtgcttctaagatcagg-3'	**Ligation**
	*Pst*I-73N-Rev	5'-**ctgcag**tccgcttccgccttctcct-3'	
	*Pst*I-73C-Fwd	5'-**ctgcag**gataaaaaaccctttccatg-3'	
	*Kpn*I-73CΔstop-Rev	5'-**ggtacc**ttcatgtggaaatggaaagg-3'	
**ΔGPE-SIIN**	*Hind*III-73N-Fwd	5'-**aagctt**ggatggtgcttctaagatcagg-3'	**Ligation**
	*Pst*I-73N-Rev	5'-**ctgcag**tccgcttccgccttctcct-3'	
	73N-*Pst*I-GE1-Fwd	5'-gaaggcggaagcgga**ctgcag**ggattaacagaattgctagg-3'	**Recombination**
	SIIN-*Pst*I-73C-Rev	5'-tgctggtaccgtcga**ctgcag**cttcatgtggaaatggaaagg-3'	
**ΔGE-SIIN**	*Pst*I-73C-Fwd	5'-**ctgcag**gataaaaaaccctttccatg-3'	**Ligation**
	*Kpn*I-73CΔstop-Rev	5'-**ggtacc**ttcatgtggaaatggaaagg-3'	
	EGFP-*Pst*I-73N-Fwd	5'-ctcaagcttcgaatt**ctgcag**ggatggtgcttctaagatcagg-3'	**Recombination**
	73C-*Pst*I-GE-GEP-Rev	5'-aaagggtttttatc**ctgcag**tacttttatatcatcttcag-3'	
**ΔE-SIIN**	EGFP-*Pst*I-73N-Fwd	5'-ctcaagcttcgaatt**ctgcag**ggatggtgcttctaagatcagg-3'	**Recombination**
	GE2-*Pst*I-GE1-Rev	5'-agttataataataac**ctgcag**ctcgtcgtcagaatctttac-3'	
	*Pst*I-73GE(E)-Fwd	5'-**ctgcag**gttattattataacttctag-3'	**Ligation**
	*Kpn*I-73CΔstop-Rev	5'-**ggtacc**ttcatgtggaaatggaaagg-3'	
**ΔGE1-SIIN**	EGFP-*Hind*III-73N-Fwd	5'-tcagatctcgagctc**aagctt**ggatggtgcttctaagatcagg-3'	**Recombination**
	SIIN-*Pst*I-GEP-Rev	5'-tgctggtaccgtcga**ctgcag**tacttttatatcatcttcag-3'	
	GEP-*Pst*I-E-Fwd	5'-gatgatataaaagta**ctgcag**ggtaaagattctgacgacgag-3'	
	SIIN-*Kpn*I-73C-Rev	5'-gaaattaattatgct**ggtacc**ttcatgtggaaatggaaagg-3'	
**ΔGE2-SIIN**	EGFP-*Hind*III-73N-Fwd	5'-tcagatctcgagctc**aagctt**ggatggtgcttctaagatcagg-3'	**Recombination**
	Cterm-*Pst*I-E-Rev	5'-aaagggtttttatc**ctgcag**gctagaagttataataataa-3'	
	*Pst*I-73C-Fwd	5'-**ctgcag**gataaaaaaccctttccatg-3'	**Ligation**
	*Kpn*I-73CΔstop-Rev	5'-**ggtacc**ttcatgtggaaatggaaagg-3'	
**ΔE-ΔGE2-SIIN**	*Pst*I-73C-Fwd	5'-**ctgcag**gataaaaaaccctttccatg-3'	**Ligation**
	*Kpn*I-73CΔstop-Rev	5'-**ggtacc**ttcatgtggaaatggaaagg-3'	
	EGFP-*Hind*III-73N-Fwd	5'-tcagatctcgagctc**aagctt**ggatggtgcttctaagatcagg-3'	**Recombination**
	73C-*Pst*I-GE1-Rev	5'- aaagggtttttatc**ctgcag**ccttgacgagtgtgagccacat-3'	
**aLANA-GEm-SIIN**	GEm-*Kpn*I-73C-Fwd	5'-gagggtagtgaggaa**ggtacc**gataaaaaaccctttccatg-3'	**Recombination**
	SIIN-*Kpn*I-73C-Rev	5'- gaaattaattatgct**ggtacc**ttcatgtggaaatggaaagg -3'	
**T7-EGFP-73Δx**	*Nhe*I-T7-*Nhe*I-EGFP-Fwd	5'-tgaaccgtcagatcc**gctagc**cccgggtaatacgactcactataggg**gctagc**g<br>ctaccggtcgccac-3'	**Recombination**
	*Nhe*I-T7-*Nhe*I-EGFP-Rev	5'-gtggcgaccggtagc**gctagc**ccctatagtgagtcgtattacccggg**gctagc**g<br>gatctgacggttca-3'	
**pHT2-aLANA/ pHT2-ΔCR/ pHT2-ΔGE**	Halo-*Nhe*I-EGFP73-Fwd	5'-tacgactcactatag**gctagc**cggactcagatctcgagctc-3'	**Recombination**
	Halo-*Nhe*I-SIIN-Rev	5'-tatccgcgccagctg**gctagc**agcttttcgaaattaattat-3'	
**GE-SIIN**	EGFP-*Hind*III-GE-Fwd	5'-tcagatctcgagctc**aagctt**ccggattaacagaattgctagg-3'	**Recombination**
	SIIN-*Hind*III-GE-Rev	5'-cgactgcagaattcg**aagctt**gaaagggtttttatcttct-3'	
**SIIN-GE-SIIN**	*Bgl*II-SIIN-*Bgl*II-73FL-Fwd	5'-tacaagtccggactc**agatct**cccggg<br>agcataattaatttcgaaaagctc**agatct**cgagctcaagcttcc-3'	**Recombination**
	*Bgl*II-SIIN-*Bgl*II-73FL-Rev	5'-ggaagcttgagctcg**agatct**gagcttttcgaaattaat<br>tatgctcccggg**agatct**gagtccggacttgta-3'	
**pEFIN3-SIIN/ pEFIN3-aLANA-SIIN/ pEFIN3-ΔGE-SIIN**	EGFP-pEFIN3-*Xba*I-Fwd	5'-ccggggatcc**tctaga**tggtgagcaagggcgag-3'	**Recombination**
	SIIN-pEFIN3-*Xba*I-Rev	5'-actagtcgac**tctaga**ttagagcttttcgaaattaatt-3'	
**pEFin3-H-2Kb**	pEFINKb-*Xba*I-Fwd	5'-ccggggatcc**tctaga**tggtaccgtgcacgctg-3'	**Recombination**
	pEFINKb-*Xba*I-Rev	5'-actagtcgac**tctaga**tcacgctagagaatgagg-3'	
