# Supplementary material for: Macavirus latency-associated protein evades immune detection through regulation of protein synthesis in cis depending upon its glycin/glutamate-rich domain
Source: PLoS Pathog. 2017 Oct 23;13(10):e1006691. doi: 10.1371/journal.ppat.1006691 (PMC5695634; doi:10.1371/journal.ppat.1006691)
Supplement: S2 Table — (PDF) [file ppat.1006691.s009.pdf]

S2 Table. Oligonucleotides used in this study to perform quantitative PCR reactions

Primer	Sequence
**ORF73-S**	5'-ggactagaccctctttatgaccc-3'
**ORF73-R**	5'-gcaatgggttcctattttgctcg-3
**OryBos-HPRT-Fwd**	5'-ggtggagatgatctctcaac-3'
**OryBos-HPRT-Rev**	5'-caacaaacttgtctggaatttc-3'
