# Supplementary material for: Macavirus latency-associated protein evades immune detection through regulation of protein synthesis in cis depending upon its glycin/glutamate-rich domain
Source: PLoS Pathog. 2017 Oct 23;13(10):e1006691. doi: 10.1371/journal.ppat.1006691 (PMC5695634; doi:10.1371/journal.ppat.1006691)
Supplement: S3 Table — (PDF) [file ppat.1006691.s010.pdf]

S3 Table. Oligonucleotides used in this study to produce BAC recombinant expression plasmids.

Primer	Sequence
**H1ΔCR-galK-Fwd**	5’-gcgggaagaaaaaaaagaaaacgaccgtaacaggagaaggcggaagcggacctgttgacaattaatcatcggca-3’
**H2ΔCR-galK-Rev**	5’- taaaagcctgatacccctgggcttcgtggacatggaaagggtttttatctcagcactgtcctgctcctt-3’
**H1ΔGE-galK-Fwd**	5’-gggaccccgaggaatcagagggaactgaagatgatataaaagtactgcagcctgttgacaattaatcatcggca -3’
**H1H2-ΔGE-Fwd**	5’-gggaccccgaggaatcagagggaactgaagatgatataaaagtactgcagga taaaaaaccctttccatgtccacgaagcccaggggtatcaggctttt -3’
**H1H2-ΔGE-Rev**	5’- aaaagcctgatacccctgggcttcgtggacatggaaagggtttttatcct gcagtacttttatatcatcttcagttccctctgattcctcggggtccc-3’
